# Does the patient with chest pain have a coronary heart disease? Diagnostic value of single symptoms and signs – a meta-analysis

**DOI:** 10.3325/cmj.2012.53.432

**Published:** 2012-10

**Authors:** Jörg Haasenritter, Damaris Stanze, Grit Widera, Christian Wilimzig, Maren Abu Hani, Andreas C Sönnichsen, Stefan Bösner, Justine Rochon, Norbert Donner-Banzhoff

**Affiliations:** 1 Department of General Practice/Family Medicine, Philipps University of Marburg, Marburg, Germany; 2 Department of Cardiology, Dr. Horst-Schmidt-Hospital, Wiesbaden, Germany; 3 Department of Family Medicine, Paracelsus University, Salzburg, Austria; 4 Institute of Medical Biometry and Informatics, University of Heidelberg, Heidelberg, Germany

## Abstract

**Aim:**

To determine the diagnostic value of single symptoms and signs for coronary heart disease (CHD) in patients with chest pain.

**Methods:**

Searches of two electronic databases (EMBASE 1980 to March 2008, PubMed 1970 to May 2009) and hand searching in seven journals were conducted. Eligible studies recruited patients presenting with acute or chronic chest pain. The target disease was CHD, with no restrictions regarding case definitions, eg, stable CHD, acute coronary syndrome (ACS), acute myocardial infarction (MI), or major cardiac event (MCE). Diagnostic tests of interest were items of medical history and physical examination. Bivariate random effects model was used to derive summary estimates of positive (pLR) and negative likelihood ratios (nLR).

**Results:**

We included 172 studies providing data on the diagnostic value of 42 symptoms and signs. With respect to case definition of CHD, diagnostically most useful tests were history of CHD (pLR = 3.59), known MI (pLR = 3.21), typical angina (pLR = 2.35), history of diabetes mellitus (pLR = 2.16), exertional pain (pLR = 2.13), history of angina pectoris (nLR = 0.42), and male sex (nLR = 0.49) for diagnosing stable CHD; pain radiation to right arm/shoulder (pLR = 4.43) and palpitation (pLR = 0.47) for diagnosing MI; visceral pain (pLR = 2.05) for diagnosing ACS; and typical angina (pLR = 2.60) and pain reproducible by palpation (pLR = 0.13) for predicting MCE.

**Conclusions:**

We comprehensively reported the accuracy of a broad spectrum of single symptoms and signs for diagnosing myocardial ischemia. Our results suggested that the accuracy of several symptoms and signs varied in the published studies according to the case definition of CHD.

Chest pain is a common complaint in all health care settings, with one of the relevant causes being coronary heart disease (CHD) (1). Despite advanced diagnostic technology being available to diagnose CHD, important first steps in the evaluation of patients with chest pain are history and physical examination. Since they allow to more appropriately identify patients in need of further investigations, they help to protect patients from harm caused by unnecessary testing and to save costs. The risk of an underlying CHD can be assessed by many symptoms, signs, and items of the medical history, each of which can be seen as a diagnostic test. Like in the case of laboratory or imaging tests, their accuracy should be assessed rigorously and corresponding recommendations should be based on the best available evidence.

The accuracy of medical history and physical examination for diagnosing CHD has been the subject of previous reviews. Mant et al (2) restricted their research question to the diagnostic value of signs and symptoms for acute coronary syndrome (ACS) and myocardial infarction (MI) in studies published until 1999. Bruyninckx et al (3) also limited the scope of their review to the outcomes ACS and MI. Furthermore, they narrowed down their review on the value of 10 pre-specified clinical symptoms and signs. Chun and McGee (4) did not restrict their research question to pre-specified symptoms, signs, or case definitions of CHD but searched only Medline. The search was conducted in 2003. Two of these reviews reported a substantial variance of results across studies but none addressed the question of potential sources of heterogeneity (2,3). None of these reviews used statistical methods currently being recommended for diagnostic accuracy reviews (5).

The aim of this study was to perform a comprehensive systematic review and quantitative meta-analysis to determine the diagnostic value of medical history and physical examination for CHD in patients with chest pain. Additionally, we explored the amount and potential sources of heterogeneity between studies.

## Methods

### Search strategy and study selection

Studies that were considered eligible were those recruiting patients presenting with acute or chronic chest pain. The target disease was CHD, with no restrictions regarding case definitions, eg, stable CHD, ACS, MI, or major cardiac event (MCE). Diagnostic tests of interest were any items of physical examination or medical history like pain characteristics, associated symptoms, cardiovascular risk factors, or history of cardiac conditions. In the following text, we refer to these items as index tests or tests. Initially, no restrictions with regard to the definition or wording of index tests were made. Studies to be included had to present data on the diagnostic value of at least one test. We considered only those tests where data for a 2 × 2 table were available in three or more studies. Otherwise, there were no restrictions in regard to the setting, study design, or study quality. However, the studies investigating solely patients with a diagnosis of CHD were excluded.

We conducted comprehensive searches of two electronic databases (EMBASE 1980 to March 2008, PubMed 1970 to August 2007). The PubMed search was updated in May 2009. Along with terms to identify chest pain and terms to identify CHD, diagnostic terms were used. Search strategies included subject headings (MeSH, Embtree) as well as free-text terms and were restricted to English and German. Detailed search strategies are available from the authors. Additionally, we searched seven relevant journals (*American Heart Journal, American Journal of Cardiology, Journal of the American College of Cardiology, Circulation, European Heart Journal, Heart, Clinical Research in Cardiology*) by hand from 1970 to June 2009 and screened the reference lists of review articles and eligible studies.

One out of three reviewers screened all identified titles and abstracts for inclusion. If uncertainty remained, full-text articles were retrieved. All potentially relevant full text articles were comprehensively assessed for eligibility. All eligible studies were reassessed by a second reviewer. Any disagreements were resolved by discussion.

### Data extraction and quality assessment

One out of three reviewers extracted relevant data, eg, bibliographic information, study design, reference disease, reference standard, definition of the index test, number of true and false positives, and true and false negatives from each study. A random sample of 10% of the data records was checked by a fourth reviewer.

Two reviewers independently assessed the study quality using the Quality of Diagnostic Accuracy Studies (QUADAS) framework (6). The categories “not clear” and “no” were combined into one. Since some of the questions referred to the index test, the assessment was made for each single index test if a study presented data on more than one test. Because items of the medical history and physical examination were the diagnostic tests of interest, question 12 (presence of clinical information for interpretation of reference tests) was omitted. In case of disagreement, the study quality was reassessed by a third reviewer.

### Analysis and data synthesis

The steps of the analysis described below were conducted separately for each index test. From the 2 × 2 tables we calculated sensitivity, specificity, and positive (pLR) and negative likelihood ratios (nLR). Additionally, we drew forest plots of sensitivity and specificity and plotted sensitivity against 1-specificity in the receiver operating characteristics (ROC) space.

We used several approaches to explore potential sources of heterogeneity and calculated the I^2^ statistic. It can be interpreted as the percentage of total variability that is due to true heterogeneity rather than sampling error (7). A special source of heterogeneity in diagnostic accuracy reviews is the so-called threshold effect, which occurs when different cut-off values are used to define test positives across primary studies. Because a negative correlation between sensitivities and specificities indicates a threshold effect, we calculated Spearman rank correlations between the logits of sensitivity and specificity (8). The R^2^ statistic was used to quantify the amount of variation that could be explained by differences in thresholds (9). If the number of studies was ≥10, we performed a meta-regression to examine whether study specific characteristics affected the results. The covariates considered in the meta-regression are listed in Table 1. Concrete definitions and categories for each covariate are provided in the supplementary Table 1.(web extra material 1)

**Table 1:** Covariates used in the meta-regression*

Criteria describing study quality:
Representative spectrum?
Acceptable reference standard?
Partial verification avoided?
Differential verification avoided?
Incorporation avoided?
Details execution index test?
Index test results blinded?
Reference standard results blinded?
Withdrawals explained?

Criteria describing clinical characteristics of patients and methodological characteristics of studies:
Reference diagnosis/ case definition of coronary heart disease
Reference standard
Setting
Patients selected?
Definite sick excluded?
Pain acute – intermediate?
Prevalence of reference disease

*For details see supplementary Table 1.(web extra material 1)

In addition to the statistical analyses, two reviewers independently assessed the forest plots and ROC curves visually (eye-balling) (10). They rated the amount of heterogeneity, the presence of a threshold effect, and the effect of the respective covariates. Any disagreements were resolved by discussion. This procedure was performed separately for each index test.

Based on the findings of the analyses of heterogeneity, we calculated summary estimates of sensitivity, specificity, and likelihood ratios using a bivariate random effects model (BREM). This approach was used because it preserves the original two-dimensional nature of the data. Using pairs of sensitivity and specificity it accounts for any possible negative correlation between these two measures. In addition, study size and between-study heterogeneity using a random effects model are taken into account (5,10,11). However, as hierarchical models are complex they do not always produce stable estimates, especially if the number of studies is small. In such cases, we presented the results of the primary studies and qualitative syntheses instead of summary estimates.

We used likelihood ratios (LR) as measures of diagnostic accuracy since they are clinically more meaningful than sensitivities, specificities, or diagnostic odds ratios. They quantify how strongly the likelihood of a disease is changed by the presence (pLR) or absence (nLR) of a symptom or sign. A LR of 1 indicates a test result without any diagnostic value. LRs of 1 to 2 or 0.5 to 1 change the likelihood very little and they are rarely important (12). LRs ≤0.5 or ≥2 change it at least moderately and can be considered as clinically helpful in the context of medical history taking and physical examination.

I^2^ statistic was calculated using MetaDiSc software (13). Meta-regressions and pooling of estimates were performed using SAS 9.2 (14), particularly the SAS macro METADAS (15). All other calculations were performed using SPSS 17 (16).

## Results

### Characteristics of included studies

**Figure 1:**
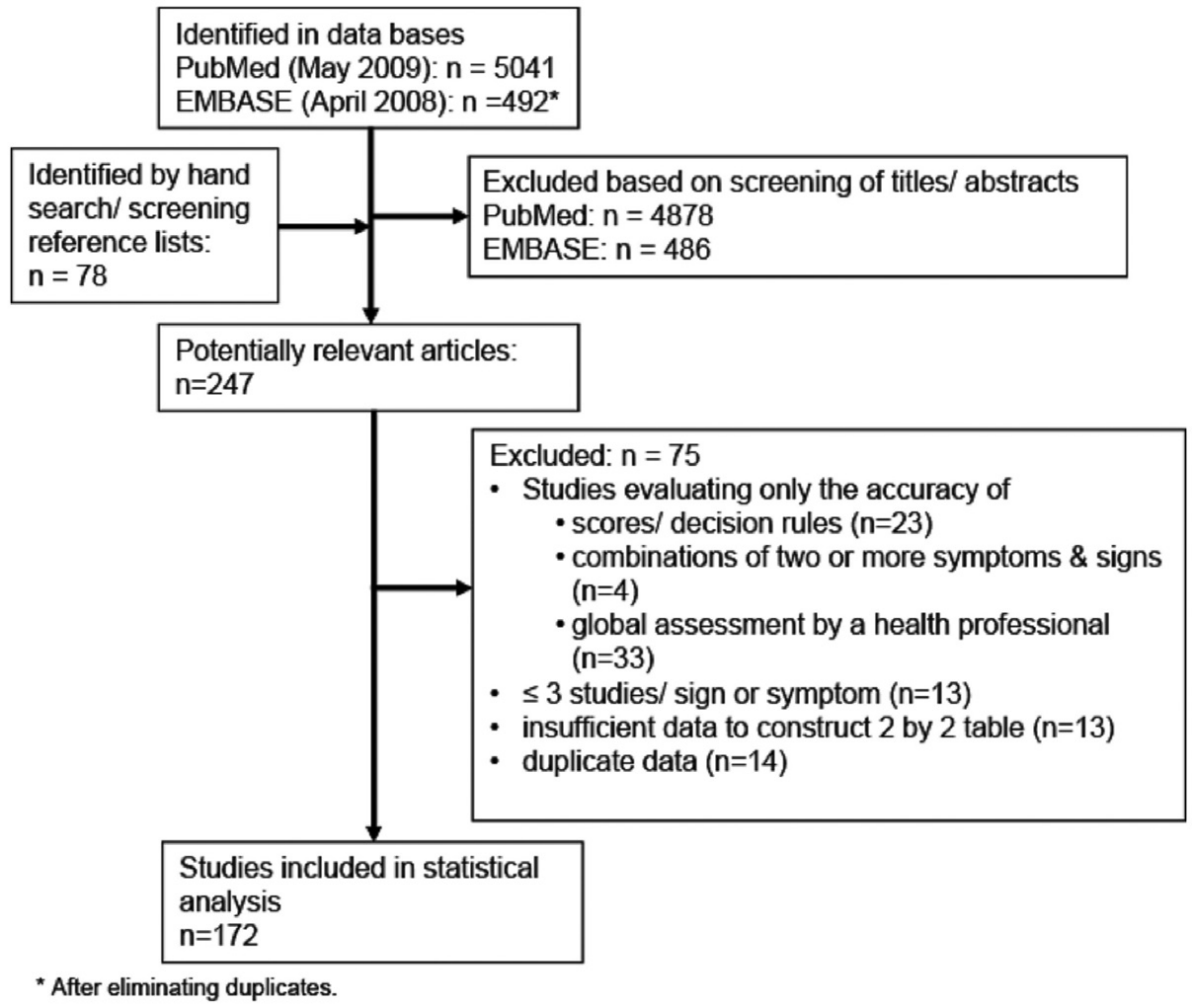
Flow of study selection.

The electronic search identified 5533 records. After having screened for inclusion criteria, we comprehensively assessed 247 full text articles (Figure 1). The range of the tests described was very broad. We combined similar symptoms or signs into one index test if appropriate (details are provided in the supplementary Table 2(web extra material 2)). In the statistical analyses we considered 42 index tests, for which at least 3 studies provided sufficient data to construct 2 × 2 tables (Table 2).

**Table 2:** Diagnostic criteria/index tests (n = 42) considered in the analyses

Cardiovascular risk factors/pre-existing cardiac conditions:
Male sex
Higher age
History of diabetes mellitus
History of dyslipidemia
History of dyslipidemia
History of hypertension
History of coronary heart disease
History of myocardial infarction
History of myocardial infarction
History of angina pectoris
Family history of myocardial infarction
Smoking
Obesity
Menopause

Pain characteristics:
Central chest pain
Left-sided chest pain
Right-sided chest pain
Radiation to left arm/shoulder
Radiation to right arm/ shoulder
Radiation to back
Visceral pain
Stabbing pain
Burning pain
Frightening pain
Time since onset of pain more than about 6 h
Typical angina pectoris
Atypical angina
Pain relief by nitro-glycerine
Crescendo angina
Pain related to breathing
Pain related to effort
Associated symptoms:
Sweating
Dyspnea
Nausea/vomiting
Dizziness
Collapse
Palpitation
Weakness
Fear/ anxiety
Physicals:
High blood pressure
Tachycardia
Bradycardia
Rales
Pain reproducible by palpation

The results of several single studies were reported in several papers. As the set of index tests sometimes varied across the papers, the final decision about duplicate data could only be made on the level of the individual index test. This resulted in 172 studies included in the analyses.

The studies were published between 1966 and 2009. The number of included patients ranged from 36 to 10 806 per study. Seventy-six percent of the included studies assessed the presence of the target disease in a consecutive series of chest pain patients and 85% were prospectively conducted (supplementary Table 3(web extra material 3)).

**Table 3:** Diagnostic accuracy of cardiovascular risk factors and pre-existing cardiac conditions*

Case definition of CHD	Studies	Patients	LR (95% CI) if RF is	
			present	absent
	**Male sex**			
Any CHD	102	99 959	1.21 (1.16-1.27)	0.70 (0.65-0.75)
Stable CHD	28	18 162	1.49 (1.35-1.63)	0.49 (0.45-0.55)
MI	44	33 776	1.12 (1.07-1.18)	0.77 (0.71-0.85)
ACS	22	33 125	1.17 (1.08-1.27)	0.79 (0.71-0.88)
MCE	8	14 896	1.13 (0.99-1.29)	0.82 (0.67-1.00)
	**Higher age**			
Any CHD	32	49 730	1.47 (1.34-1.60)	0.70 (0.61-0.80)
Stable CHD	7	10 804	1.49 (1.24-1.80)	0.63 (0.47-0.85)
MI	15	20 180	1.41 (1.25-1.59)	0.76 (0.65-0.89)
ACS	7	13 597	1.44 (1.19-1.73)	0.69 (0.52-0.92)
MCE	3	5149	1.75 (1.26-2.44)	0.55 (0.33-0.93)
	**History of diabetes mellitus**			
Any CHD	72	74 418	1.67 (1.47-1.90)	0.91 (0.89-0.93)
Stable CHD	30	16 371	2.16 (1.81-2.58)	0.87 (0.84-0.90)
MI	21	18 139	1.18 (0.97-1.43)	0.97 (0.95-1.00)
ACS	15	27 429	1.68 (1.35-2.09)	0.89 (0.85-0.94)
MCE	6	12 479	1.85 (1.30-2.64)	0.87 (0.79-0.95)
	**History of dyslipidemia**			
Any CHD	46	41 377	1.52 (1.34-1.73)	0.73 (0.67-0.80)
Stable CHD	23	15 908	1.53 (1.30-1.80)	0.68 (0.60-0.76)
MI	7	6331	1.20 (0.89-1.62)	0.88 (0.73-1.06)
ACS	12	15 634	1.62 (1.24-2.11)	0.76 (0.66-0.87)
MCE	4	3504	1.72 (1.07-2.75)	0.75 (0.59-0.95)
	**History of hypertension**			
Any CHD	70	61 858	1.30 (1.19-1.43)	0.83 (0.78-0.89)
Stable CHD	33	19 241	1.30 (1.16-1.46)	0.80 (0.72-0.88)
MI	19	16 077	1.10 (0.91-1.33)	0.96 (0.88-1.04)
ACS	14	17 185	1.55 (1.27-1.89)	0.72 (0.62-0.84)
MCE	4	9355	1.39 (0.98-1.98)	0.78 (0.58-1.04)
	**History of CHD**			
Any CHD	65	66 364	1.47 (1.25-1.74)	0.83 (0.77-0.90)
Stable CHD	13	4615	3.59 (2.63-4.90)	0.59 (0.51-0.68)
MI	32	28 077	0.97 (0.83-1.13)	1.01 (0.94-1.09)
ACS	15	29 616	1.70 (1.36-2.13)	0.77 (0.68-0.86)
MCE	5	4056	1.65 (1.09-2.50)	0.80 (0.66-0.98)
	**History of MI**			
Any CHD	52	47 056	1.37 (1.14-1.64)	0.87 (0.80-0.95)
Stable CHD	11	3234	3.21 (2.34-4.40)	0.57 (0.49-0.67)
MI	30	26 281	0.93 (0.80-1.09)	1.03 (0.96-1.10)
ACS	7	16 477	1.70 (1.24-2.33)	0.80 (0.70-0.91)
MCE	4	1064	1.42 (0.93-2.16)	0.82 (0.65-1.04)
	**History of angina pectoris**			
Any CHD	22	25 761	1.09 (0.92-1.30)	0.93 (0.79-1.09)
Stable CHD	2	490	1.40 (1.01-1.94)	0.42 (0.23-0.77)
MI	15	12 541	0.89 (0.77-1.03)	1.08 (0.98-1.20)
ACS	3	12 017	1.92 (1.37-2.68)	0.57 (0.42-0.77)
MCE	2	713	1.06 (0.80-1.42)	0.91 (0.59-1.40)
	**Family history of MI**			
Any CHD	34	28 060	1.19 (1.06-1.33)	0.90 (0.85-0.96)
Stable CHD	18	7403	1.07 (0.94-1.21)	0.95 (0.86-1.04)
MI	5	6397	1.20 (0.92-1.56)	0.92 (0.81-1.04)
ACS	8	13 748	1.44 (1.14-1.83)	0.84 (0.76-0.94)
MCE	3	512	1.44 (0.90-2.30)	0.88 (0.73-1.05)
	**Smoking**			
Any CHD	68	55 164	1.28 (1.20-1.37)	0.87 (0.82-0.91)
Stable CHD	34	19 512	1.38 (1.26-1.51)	0.82 (0.77-0.88)
MI	16	9900	1.24 (1.10-1.40)	0.85 (0.76-0.95)
ACS	13	16 265	1.15 (0.96-1.37)	0.95 (0.88-1.02)
MCE	5	9487	1.06 (0.82-1.37)	0.96 (0.82-1.13)
	**Obesity**			
Any CHD	12	9852	1.06 (0.88-1.26)	0.97 (0.89-1.07)
Stable CHD	7	8062	1.07 (0.89-1.30)	0.95 (0.83-1.09)
MI	2	451	0.95 (0.62-1.46)	1.03 (0.81-1.32)
ACS	2	1219	1.03 (0.59-1.80)	0.99 (0.88-1.12)
MCE	1	120	1.11 (0.36-3.38)	0.98 (0.79-1.22)

*Abbreviations: CHD – coronary heart disease; MI – myocardial infarction; ACS – acute coronary syndrome; MCE – major cardiac event; LR – likelihood ratio; RF – risk factor; CI – confidence interval.

### Heterogeneity and subgroup analyses

I^2^ ranged from 0 to 98.6% and was above 80% in 40 of the analyzed index tests, indicating a substantial amount of between-study heterogeneity (supplementary Table 4(web extra material 4)). In 22 tests, we found evidence of a threshold effect, indicated by a significant negative correlation between the logits of sensitivity and specificity. R^2^ ranged from 0.21 to 1.00, indicating the proportion of variance that can be explained by differences in thresholds (supplementary Table 5(web extra material 5)).

**Table 4:** Diagnostic accuracy of pain characteristics

Case definition of CHD	Studies	Patients	LR (95% CI) if symptom is	
			present present	absent
	**Central chest pain**			
Any CHD	14	11 673	1.26 (1.14-1.40)	0.69 (0.53-0.92)
Stable CHD	5	885	1.36 (1.09-1.69)	0.65 (0.38-1.10)
MI	9	10 788	1.23 (1.10-1.38)	0.71 (0.50-0.99)
	**Left-sided chest pain**			
Any CHD^†^	12	5608	0.85 (0.60-1.20)	1.06 (0.96-1.18)
	**Right-sided chest pain**			
Any CHD	5	2170	1.06 (0.55-2.05)	0.99 (0.86-1.14)
Stable CHD	2	535	0.76 (0.51-1.14)	1.17 (0.81-1.68)
MI	3	1635	1.39 (0.58-3.34)	0.96 (0.87-1.06)
	**Radiation to left arm/shoulder**			
Any CHD^†^	12	16 316	1.30 (1.12-1.52)	0.86 (0.78-0.95)
	**Radiation to right arm/shoulder**			
Any CHD	9	2717	2.95 (1.42-6.12)	0.87 (0.80-0.96)
Stable CHD	3	627	1.42 (0.66-3.03)	0.93 (0.82-1.05)
MI	6	2090	4.43 (1.77-11.10)	0.87 (0.77-0.97)
	**Visceral pain**			
Any CHD	17	15 059	1.34 (1.07-1.67)	0.78 (0.60-1.02)
Stable CHD	5	885	1.34 (1.01-1.78)	0.68 (0.37-1.22)
MI	10	13194	1.21 (0.89-1.63)	0.87 (0.66-1.15)
ACS	2	980	2.05 (1.14-3.68)	0.63 (0.28-1.43)
	**Stabbing pain**			
Any CHD	11	12 269	0.86 (0.48-1.56)	1.02 (0.94-1.10)
Stable CHD	4	939	0.90 (0.41-2.00)	1.02 (0.87-1.19)
MI	6	11082	0.69 (0.34-1.40)	1.04 (0.94-1.15)
ACS	1	248	3.65 (0.45-29.94)	0.93 (0.83-1.04)
	**Burning pain**			
Any CHD	7	2582	1.38 (0.93-2.04)	0.97 (0.93-1.01)
Stable CHD	2	535	1.51 (0.74-3.08)	0.96 (0.90-1.03)
MI	5	2047	1.35 (0.87-2.09)	0.97 (0.93-1.02)
	**Frightening pain**			
Any CHD^†^	4	1608	1.40 (0.61-3.21)	0.92 (0.79-1.08)
	**Time since onset of pain >6 h**			
Any CHD	9	14 499	0.85 (0.66-1.09)	1.09 (0.95-1.26)
MI	6	12 212	0.82 (0.59-1.14)	1.10 (0.93-1.29)
ACS	3	2287	0.90 (0.62-1.30)	1.08 (0.82-1.44)
	**Typical angina**			
Any CHD	14	28 482	2.36 (1.47-3.79)	0.61 (0.44-0.86)
Stable CHD	13	19 720	2.35 (1.44-3.83)	0.61 (0.43-0.87)
MCE	1	8762	2.60 (0.46-14.63)	0.58 (0.17-2.06)
	**Atypical angina**			
Any CHD	5	19 524	0.72 (0.53-0.97)	1.28 (1.01-1.62)
Stable CHD	4	10 762	0.74 (0.53-1.05)	1.22 (0.96-1.57)
MCE	1	8762	0.64 (0.37-1.11)	1.53 (0.87-2.67)
	**Pain relief by nitro-glycerine**			
Stable CHD	9	2813	1.25 (1.05-1.48)	0.77 (0.65-0.92)
	**Pain related to effort**			
Any CHD	8	2975	1.89 (1.23-2.92)	0.74 (0.56-0.97)
Stable CHD	6	1302	2.13 (1.36-3.33)	0.67 (0.49-0.91)
MI	2	1673	1.22 (0.50-2.96)	0.94 (0.69-1.28)

*Abbreviations: CHD – coronary heart disease; MI – myocardial infarction; ACS – acute coronary syndrome; MCE – major cardiac event; LR – likelihood ratio; CI – confidence interval.

†Results for different case definitions of CHD not provided, since the random effects meta-analysis with covariate “case definition” did not produce stable estimates.

**Table 5:** Diagnostic accuracy of associated symptoms and physical examination

Case definition of CHD	Studies	Patients	LR (95% CI) if symptom or sign is	
			present	absent
	**Sweating**			
Any CHD^†^	11	16 011	2.05 (1.73-2.42)	0.73 (0.61-0.87)
	**Dyspnea**			
Any CHD	20	27 322	0.86 (0.77-0.96)	1.08 (1.01-1.14)
Stable CHD	6	2914	0.95 (0.78-1.15)	1.04 (0.91-1.18)
MI	10	11 939	0.89 (0.76-1.03)	1.06 (0.98-1.15)
ACS	4	12 469	0.72 (0.56-0.93)	1.14 (0.99-1.30)
	**Nausea/vomiting**			
Any CHD^†^	13	16 082	1.54 (1.32-1.79)	0.83 (0.75-0.92)
	**Dizziness**			
Any CHD^†^	7	11 456	0.59 (0.52-0.67)	1.13 (1.04-1.22)
	**Collapse**			
Any CHD^†^	9	13 870	0.53 (0.35-0.81)	1.06 (1.00-1.13)
	**Palpitations**			
Any CHD	10	5187	0.55 (0.38-0.79)	1.11 (1.01-1.22)
Stable CHD	3	809	0.66 (0.37-1.18)	1.04 (0.95-1.13)
MI	5	2588	0.47 (0.28-0.81)	1.12 (0.98-1.27)
ACS	2	1790	0.61 (0.27-1.37)	1.04 (0.95-1.13)
	**Weakness**			
Any CHD^†^	6	4611	1.20 (0.97-1.48)	0.90 (0.78-1.04)
	**Rales**			
Any CHD^†^	8	19 700	1.81 (1.03-3.17)	0.88 (0.81-0.95)

*Abbreviations: CHD – coronary heart disease; MI – myocardial infarction; ACS – acute coronary syndrome; LR – likelihood ratio; CI – confidence interval.

†Results for different case definitions of CHD not provided, since the model with covariate “case definition” did not produce stable estimates.

In the meta-regression, no covariate showed a consistent effect on the diagnostic accuracy. Among the covariates, reference diagnosis/case definition of CHD most frequently influenced the accuracy of an index test (supplementary Table 6(web extra material 6)). Therefore, we decided to provide pooled estimates across all studies and additionally within each subgroup determined by the case definition of CHD. The reference diagnosis or case definition of CHD used was ACS in 17%, MI in 43%, stable CHD in 35%, and MCE in 5% of the included studies. If ACS or MI was the target disease, the majority of patients presented with acute chest pain and the reference diagnosis was established by a combination of clinical presentation, biomarker elevations, and ECG changes (supplementary Table 3(web extra material 3)). ACS comprised ST elevation myocardial infarction, non-ST elevation myocardial infarction, and unstable angina. The first included study using this case definition as reference diagnosis was published in 1995 (17). If stable CHD was the target disease, the majority of patients presented with chronic/intermediate chest pain. In 74% of these 60 studies, the diagnosis was established using coronary angiography (supplementary Table 3(web extra material 3)). The outcome MCE comprised combinations of different outcomes such as MI, ventricular tachycardia, and revascularization. The prevalence of CHD with respect to the case definition of CHD ranged from 8.1 to 79.7% (ACS), from 3.8 to 88.0 (MI), from 16.7 to 89.9 (stable CHD), and from 2.8 to 34.1% (MCE).

**Figure 2:**
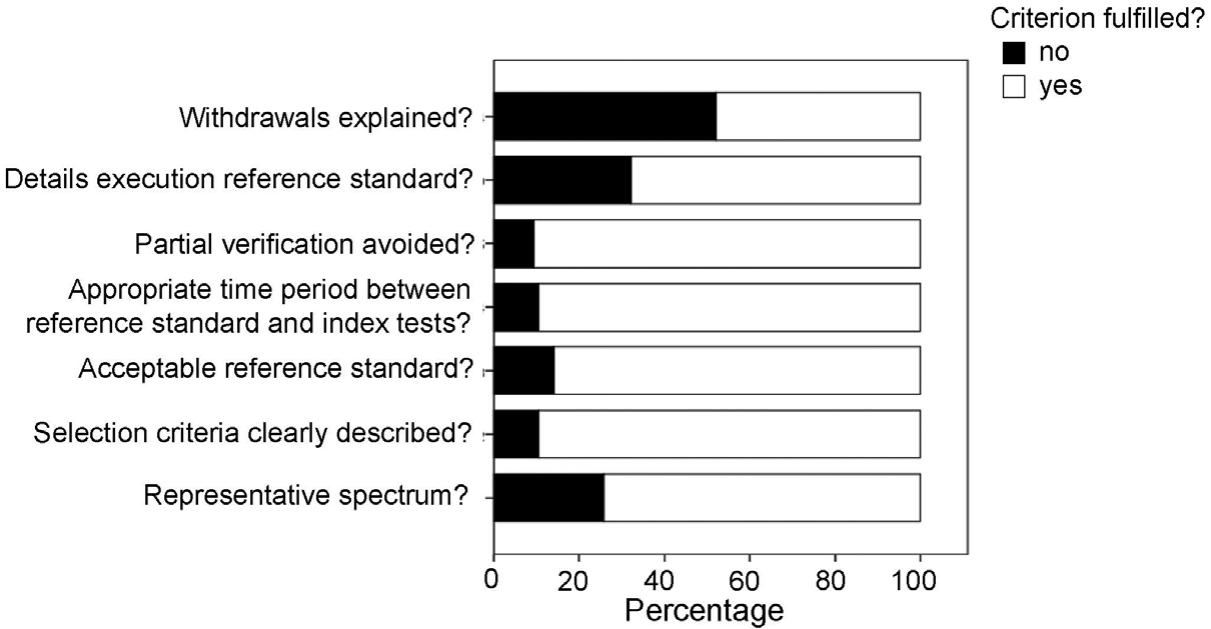
Methodological quality of included studies in regard to 7 quality criteria.

Out of the 13 QUADAS items, only 7 referred to the whole study. Considering these items, the quality of the included studies was fair (Figure 2). The other 6 items can only be applied to an individual index test. In 8 index tests, we found evidence that one or two of the quality criterions considered as covariates were significantly associated with the accuracy of an index test (supplementary Table 6(web extra material 6)). In that case, we calculated pooled estimates of pLR and nLR within the respective subgroups (supplementary Table 7(web extra material 7)).

### Diagnostic value of symptoms and signs

*Cardiovascular risk factors and pre-existing cardiac conditions.* Twelve index tests referred to the presence or absence of cardiovascular risk factors and pre-existing cardiac conditions. The number of studies per index test ranged from 5 (menopause) to 102 (male sex) (median: 49). For 11 of these index tests, a quantitative synthesis was possible across all studies and within subgroups. For the diagnosis of myocardial ischemia in general, pLRs ranged from 1.06 (obesity) to 1.67 (history of diabetes), and nLRs ranged from 0.70 (age, sex) to 0.97 (obesity). However, accuracy varied across subgroups determined by case definition of CHD. Within the stable CHD subgroup, the most helpful diagnostic criteria were history of diabetes (pLR = 2.16), history of CHD (pLR = 3.59), and history of MI (pLR = 3.21). In the MI subgroup, the confidence interval of the positive and negative LRs of 8 index tests included “1,” indicating that absence or presence of these findings did not significantly change the likelihood of CHD as an underlying cause (Table 3).

Quantitative synthesis was not possible for one risk factor (menopause). Results of pLR and nLR in the five primary studies ranged from 1.11 to 1.18, and 0.62-0.75, respectively (supplementary Table 8(web extra material 8)).

*Pain characteristics.* Seventeen index tests referred to pain characteristics such as localization or radiation, pain quality, time of onset, and provoking or revealing factors. The number of studies per index test ranged from 3 (crescendo angina, pain related to breathing) to 17 (visceral pain) (median: 9).

In 14 index tests quantitative syntheses across all studies were possible, and in 10 index tests subgroup analyses in regard to case definition of CHD (Table 4). For the diagnosis of myocardial ischemia in general, most helpful pain characteristics were the presence of pain radiation to right arm/shoulder (pLR = 2.95) and typical angina (pLR = 2.36). In all other index tests, positive and negative LRs ranged between 0.5 and 2.0. In two index tests, the accuracy varied substantially across subgroups determined by the case definition of CHD. In pain radiation to right arm/shoulder, pLR was 4.43 if MI was the target disease and 1.42 if stable CHD was the target disease. The presence of stabbing pain showed a pLR of 3.65 if ACS was the target disease, 0.90 for stable CHD, and 0.69 for MI. However, only one study contributed to the diagnostic outcome ACS (18). The 95% confidence interval ranged from 0.45 to 29.94 and only 10 out of 248 patients presented with stabbing pain. In three index tests (left-sided chest pain, radiation to left arm/shoulder, frightening pain), quantitative syntheses across all studies were done but no subgroup analyses were possible. The qualitative subgroup analyses showed similar between- and within-subgroup variation (supplementary Table 9(web extra material 9)). In further three index tests (radiation to back, crescendo angina, and pain related to breathing), only qualitative syntheses were possible. Results showed that pain related to breathing might be helpful for ruling out myocardial ischemia, with pLR ranging from 0.20 to 0.36 in primary studies.

*Associated symptoms and signs.* Eight index tests referred to the presence or absence of associated symptoms. The number of studies per index test ranged from 3 (fear/anxiety) to 20 (dyspnea) (median: 9.5). A quantitative synthesis across all studies to determine the accuracy of the diagnosis of myocardial ischemia in general was possible in 7 index tests (Table 5). The most helpful symptom was the presence of sweating (pLR = 2.05). In most index tests, variation between subgroups was similar to the variation within subgroups (supplementary Table 10(web extra material 10)). In the index test palpitations, the pLR was 0.47 for the diagnosis of MI, 0.61 for ACS, and 0.66 for stable CHD. In the index test collapse studies using the diagnostic outcome, ACS showed a pLR ranging from 0.25 to 0.42. In contrast, if MI was the diagnostic outcome pLRs ranged from 0.66 to 1.82.

Five index tests referred to results of the physical examination. The number of studies per index test ranged from 3 (high blood pressure, tachycardia, bradycardia) to 8 (rales, pain reproducible by palpation) (median: 3). A quantitative synthesis across all studies to determine the accuracy for the diagnosis of myocardial ischemia in general was only possible in one index test (Table 5). Results of the qualitative subgroup analyses showed that pain reproducible by palpation might be helpful for ruling out myocardial ischemia, with pLR ranging from 0.13 to 0.41 in primary studies (supplementary Table 11(web extra material 11)). Noticeably, one study showed extreme values for the presence of tachycardia (pLR: 20.50), bradycardia (pLR:13.04), and rales (pLR: 13.70) (19).

## Discussion

In this study, we investigated the accuracy of single symptoms and signs for CHD in patients with chest pain. Overall, the presence of clinical findings was more informative than the absence. Most helpful for the diagnosis of myocardial ischemia in general was the presence of typical angina, radiation of pain to the right arm/shoulder, pain reproducible by palpation, and pain related to breathing. However, in several index tests we found that the accuracy varied across subgroups determined by case definition of CHD. In respect to the case definition, diagnostically most useful tests were history of CHD (pLR = 3.59), known MI (pLR = 3.21), typical angina (pLR = 2.35), history of diabetes mellitus (pLR = 2.16), exertional pain (pLR = 2.13), history of angina pectoris (nLR = 0.42), and male sex (nLR = 0.49) for diagnosing stable CHD; pain radiation to right arm/shoulder (pLR = 4.43), and palpitation (pLR = 0.47) for diagnosing MI; visceral pain (pLR = 2.05) for diagnosing ACS, and typical angina (pLR = 2.60) and pain reproducible by palpation (pLR = 0.13) for predicting MCE.

About 60 studies included in this review were published after Chun and McGee (4) had made their search, indicating that it was reasonable to conduct a new review. A recently published review on the diagnostic value of nitro-glycerine included 5 studies with 1978 patients (20). We identified 4 additional studies including 835 patients. This may be an indication that our more comprehensive search strategy without restriction on pre-specified symptoms and signs resulted in high sensitivity.

We used the BREM approach to calculate pooled estimates. Since this model is rather complex it sometimes fails to converge or produce stable estimates, especially if the number of studies is small. As a consequence we could not present pooled estimates for several index tests across all studies and/or within subgroups. However, it was mandatory to use the BREM since only hierarchical models like the BREM or the hierarchical summary ROC model account for the different sources of heterogeneity we identified in the analyses (5).

We separately determined the accuracy of each single symptom and sign. This approach did not account for dependence between the respective index tests and might have resulted in a biased estimation of the measures of accuracy. However, considering dependence between the index tests would require a multivariate approach using individual patient data (21,22).

Current guidelines recommend that the clinician should consider items of the medical history like age, sex, characteristics of pain like localization, radiation, and quality, associated symptoms, cardiovascular risk factors, and pre-existing cardiac conditions in the diagnosis of CHD in patients with chest pain (23,24). The symptoms and signs considered in our review represented all these categories. Not surprisingly, none of these clinical findings showed in isolation a sufficient diagnostic accuracy for safely ruling in or ruling out myocardial ischemia in patients presenting with chest pain.

Assessment of cardiovascular risk profile is supposed to be a corner stone in the clinical evaluation of patients with chest pain. However, in our study the diagnostic value of single cardiovascular risk factors was low to moderate. This is not surprising. Wald et al (25) pointed out that the association between a risk factor and a disease must be very strong before the risk factor could be considered as a worthwhile test. The accuracy of most single cardiovascular risk factors was higher when estimating the likelihood of a stable CHD compared to ACS, MI, or MCE. Most noticeably, information on the presence of most single cardiovascular risk factors was diagnostically useless when estimating the likelihood of MI. This seems to be plausible if one considers that cardiovascular risk factors are supposed to predict the development of a chronic disease rather than the acute manifestation of the disease.

The diagnostically most helpful symptom describing the localization and radiation of pain was the right-sided radiation of pain. The accuracy was highest for the diagnostic outcome MI. This finding was consistent with previous reviews (2,3). When estimating the likelihood of stable CHD, the most informative symptom was pain described as typical angina or pain related to effort. These classical features remain the most persuasive findings arguing for a diagnosis of stable CHD (26).

Among the associated symptoms, the presence of sweating and nausea/vomiting showed a consistent but small effect when estimating the likelihood of MI in the majority of studies. The most helpful finding arguing against myocardial ischemia in general was pain that was reproducible by palpation. However, these findings could only be based on the qualitative syntheses.

One study showed remarkable results for the accuracy of three physical findings (tachycardia, bradycardia, rales) (19). Among the studies investigating the accuracy of these index tests, this study was the only one conducted in primary care. The difference might be a hint toward a spectrum effect. The proportion of patients without an underlying coronary ischemia but with an underlying serious cardiac condition and correspondent findings might be higher in secondary than in primary care (27). Accordingly, the accuracy of findings might differ between settings. Because the number of primary care studies was extremely small we could not systematically address this issue.

Medical history taking and physical examination are crucial steps in the evaluation of patients with chest pain. In this study, we reported the accuracy of a broad spectrum of symptoms and signs for myocardial ischemia as the underlying reason. Our results also suggested that the accuracy of several symptoms and signs varied in the published papers according to the case definition of CHD.
