# Supplementary material for: Does the patient with chest pain have a coronary heart disease? Diagnostic value of single symptoms and signs – a meta-analysis
Source: Croat Med J. 2012 Oct;53(5):432–41. doi: 10.3325/cmj.2012.53.432 (PMC3490454; doi:10.3325/cmj.2012.53.432)
Supplement: Supplementary Table 1 [file CroatMedJ_53_s001.pdf]

Supplemental table 1: Covariates considered in meta-regression

Covariate	Definition	Values
Criteria describing study quality		
Representative spectrum?	Was the spectrum of patients representative of the patients who will receive the test in practice?<br>Refers to QUADAS item No 1.	• Yes<br>• No or unclear
Acceptable reference standard?	Is the reference standard likely to correctly classify the target condition?<br>Refers to QUADAS item No 3.	• Yes<br>• No or unclear
Partial verification avoided?	Did the whole sample or a random selection of the sample, receive verification using a reference standard of diagnosis?<br>Refers to QUADAS item No 5.	• Yes<br>• No or unclear
Differential verification avoided?	Did patients receive the same reference standard regardless of the index test result?<br>Refers to QUADAS item No 6.	• Yes<br>• No or unclear
Incorporation avoided?	Was the reference standard independent of the index test (i.e. the index test did not form part of the reference standard)?<br>Refers to QUADAS item No 7.	• Yes<br>• No or unclear
Details execution index test?	Was the execution of the index test described in sufficient detail to permit replication of the test?<br>Refers to QUADAS item No 8.	• Yes<br>• No or unclear
Index test results blinded?	Were the index test results interpreted without knowledge of the results of the reference standard?<br>Refers to QUADAS item No 10.	• Yes<br>• No or unclear
Reference standard results blinded?	Were the reference standard results interpreted without knowledge of the results of the index test?<br>Refers to QUADAS item No 11.	• Yes<br>• No or unclear
Withdrawals explained?	Were withdrawals from the study explained?<br>Refers to QUADAS item No 14.	• Yes<br>• No or unclear
Criteria describing clinical characteristics of patients and study methods		
Reference diagnosis/ Case definition	Case definition of coronary heart disease used in the study.	• Coronary heart disease<br>• Acute coronary syndrome<br>• Myocardial infarction<br>• Major cardiac event
Reference	Reference standard used in the study to establish the	Coronary

standard	reference diagnosis.	angiography<br>Cardiac computed tomography<br>• Perfusion scintigraphy<br>• Magnetic resonance imaging<br>• Combination (e.g. clinical findings, ECG findings, cardiac enzymes)<br>• Clinical course<br>• Cardiac enzymes
Setting	In which setting was the study conducted?	• Hospital<br>• practice<br>• paramedics<br>• not reported
Patients selected?	Were patients preselected by a clinician based on the probability of coronary heart disease before recruitment?	• yes<br>• no<br>• mixed<br>• not reported
Definite sick excluded?	Were patients excluded if the diagnosis of coronary heart disease was obvious at time of recruitment?	• yes<br>• no<br>• not reported
Pain acute – intermediate?	Did the included patients present with acute or intermediate/ chronic chest pain?	• acute<br>• intermediate/ chronic<br>• mixed<br>• not reported
Prevalence of reference disease	Was the prevalence of the disease low, medium, or high? For each reference diagnosis separate strata were defined.<br><br>Coronary heart disease / Myocardial infarction/ acute coronary syndrome<br>low: < 20 % / < 6 %<br>medium: 20-50 % / 6-15%<br>high: > 50 % / > 15 %	• low<br>• medium<br>• high<br>• not reported
Study size	≤200	

	>200	
Date of publication	<1998<br>≥1998	
