# Supplementary material for: Does the patient with chest pain have a coronary heart disease? Diagnostic value of single symptoms and signs – a meta-analysis
Source: Croat Med J. 2012 Oct;53(5):432–41. doi: 10.3325/cmj.2012.53.432 (PMC3490454; doi:10.3325/cmj.2012.53.432)
Supplement: Supplementary Table 2 [file CroatMedJ_53_s002.pdf]

Supplemental table 2: Diagnostic criteria/ index tests considered in the analyses

| Clinical finding | Definitions used in primary studies/ Annotations |
|---|---|
| Cardiovascular risk factors and pre-existing cardiac conditions | |
| Male sex | |
| Higher age | Included studies used different thresholds. If more than 2 categories were presented we used a value of about 65 as threshold to dichotomize the data. |
| History of diabetes mellitus | |
| History of dyslipidaemia | Hypercholestemia, Dyslipidemia, Hyperlipidemia/ Only nine studies provided exact thresholds. |
| History of hypertension | |
| History of CHD | History of MI, history of angina pectoris, history of CHD, history of CABG, history of PCI. If one study provided data on more than one definition we used the data in following order: MI, CHD, history of CABG or PCI, history of angina pectoris. |
| History of MI | |
| History of Angina pectoris | |
| Family history of MI | |
| Smoking | Smoking, current smoking, history of smoking, smoking in the past or present. |
| Obesity | |
| Menopause | |
| Pain characteristics | |
| Central chest pain | Substernal pain, retrosternal pain, pain localized in upper middle or upper central area of chest. |
| Left-sided chest pain | parasternal left, pain left of sternum, left anterior chest pain, site of pain: left, pain localization: upper left. |
| Right-sided chest pain | Parasternal right, middle right, site of pain: right. |
| Radiation to left arm/ shoulder | Radiation to left arm/ left shoulder. |
| Radiation to right arm/ shoulder | Radiation to right arm/ right shoulder. |
| Radiation to back | |
| Visceral pain | Pain described as crushing, pressing, dull, heavy, oppressive, or, gripping. |
| Stabbing pain | Pain quality or character described as sharp, stabbing, cutting, one-point. |
| Burning pain | Pain quality or character described as burning. |
| Frightening pain | Pain quality or character described as frightening. |
| Time since onset of pain more than about 6h | Duration of pain > 6h, duration of pain onset>6h, arrival at emergency department>6h after pain onset, time since pain begin>4h, symptom duration > 6h. |
| Typical angina | Pain characterized as typical or definite angina, characteristic distress |

| | |
|---|---|
| | in chest was consistently precipitated by effort and relieved within minutes by rest or treatment with nitro-glycerine |
| Atypical angina | |
| Pain relief by nitro-glycerine | Chest pain relief by nitro, relief with nitro<5 min,. |
| Crescendo angina | Accelerating pain, crescendo angina. |
| Pain related to breathing | Pain reproduced by deep breathing/ inspiration. |
| Pain related to effort | Exertional pain, effort angina, angina induced by exercise, pain on effort. |
| Associated symptoms | |
| Sweating | Diaphoresis, sweating, cold sweat |
| Dyspnoea | Dyspnoea, shortness of breath, breathlessness |
| Nausea/ vomiting | Nausea, vomiting, Nausea and vomiting. If data on both symptoms were separately presented we used the data provided for vomiting. |
| Dizziness | Dizziness, faintness, vertigo. |
| Collapse | Syncope, unconsciousness, fainting. |
| Palpitation | Palpitation, feeling of arrhythmia. |
| Weakness | Weakness, fatigue, tiredness. |
| Fear/ anxiety | Feeling of anxiety, fear of death, distress. |
| Physicals | |
| High blood pressure | Blood pressure > 160/ 95, blood pressure > 180. |
| Tachycardia | Heart rate>100 bpm, heart rate>120 bpm. |
| Bradycardia | Heart rate < 50bpm, heart rate<60bpm. |
| Rales | Rales |
| Pain reproducible by palpation, tenderness | Chest wall tenderness, Chest pain reproduced by chest wall palpation. |

| | |
|---|---|
| Bpm | Beats per minute |
| CABG | Coronary artery bypass graft |
| CHD | Coronary heart disease |
| MCE | Major cardiac event |
| MI | Myocardial infarction |
| PCI: | Percutaneous coronary intervention |
