# Supplementary material for: Does the patient with chest pain have a coronary heart disease? Diagnostic value of single symptoms and signs – a meta-analysis
Source: Croat Med J. 2012 Oct;53(5):432–41. doi: 10.3325/cmj.2012.53.432 (PMC3490454; doi:10.3325/cmj.2012.53.432)
Supplement: Supplementary Table 3 [file CroatMedJ_53_s003.pdf]

Supplemental table 3: Characteristics of included studies

Study		Country	Design		Size (n)	Reference disease	Reference standard	Patients with disease (%)
McConahay 1971	1	USA	consecutive	prospective	100	CHD	Angiography	65.0
Sawe 1971	2	S	consecutive	nr	137	MI	Combination	38.6
Sawe 1972	3	S	consecutive	retrospective	1046	MI	Combination	48.9
Sawe 1972a	4	S	consecutive	prospective	191	MI	Combination	56.0
Piessens 1974	5	USA	nr	nr	70	CHD	Angiography	57.1
Detry 1977	6	B	nr	nr	278	CHD	Angiography	73.4
Schroeder 1978	7	USA	consecutive	prospective	211	MI	Combination	43.1
Waters 1978	8	CDN	not consecutive	prospective	239	CHD	Angiography	43.5
Plotnick 1979	9	USA	consecutive	prospective	200	CHD	Angiography	17.5
Schroeder 1979	10	USA	consecutive	prospective	211	MI	Combination	43.1
Pearson 1979	11	USA	nr	retrospective	483	CHD	Angiography	71.6
van der Does 1980	12	NL	consecutive	prospective	1343	MI	Combination	9.7
Grande 1980	13	DK	consecutive	prospective	401	MI	Combination	47.9
Short 1981	14	GB	consecutive	prospective	383	MI	Combination	40.0
Holmes 1981	15	USA	consecutive	prospective	431	CHD	Angiography	82.6
Chaitman 1981	16	CDN	consecutive	retrospective	8157	CHD	Angiography	55.2
Craig 1982	17	AUS	consecutive	prospective	380	MI	Combination	57.1
Kambara 1982	18	J	nr	prospective	59	CHD	Angiography	62.7
Hickam 1982	19	USA	consecutive	prospective	241	CHD	Angiography	75.8
Beunderman 1983	20	NL	not consecutive	retrospective	106	MI	Clinical course	50.0
Melin 1983	21	B	consecutive	prospective	224	CHD	Angiography	79.5
Sirnes 1984	22	N	consecutive	prospective	227	MI	Enzymes	70.7
Lee 1985	23	USA	consecutive	prospective	596	MI	Combination	17.4
Turi 1985	24	USA	consecutive	prospective	903	MI	Combination	88.0
Brown 1985	25	GB	nr	prospective	101	MI	Clinical course	53.5
Davies 1985	26	GB	consecutive	prospective	80	CHD	Combination	52.0
Underwood 1985	27	USA	not consecutive	retrospective	176	CHD	Angiography	50.0
Joswig 1985	28	USA	consecutive	prospective	184	CHD	Angiography	58.2
Logan 1986	29	NZ	consecutive	prospective	227	MI	Combination	43.2
Tierney 1986	30	USA	consecutive	prospective	492	MI	Combination	12.4
Toyosaki 1986	31	J	nr	prospective	1000	CHD	Combination	24.8
Kladetzky 1986	32	D	nr	prospective	497	CHD	Angiography	66.6
Madsen 1987	33	DK	consecutive	prospective	532	MI	Combination	51.7
Hedges 1987	34	USA	consecutive	prospective	773	MI	Combination	6.6
Miller 1987	35	USA	consecutive	prospective	100	MI	Enzymes	43.0
Herlihy 1987	36	USA	consecutive	prospective	265	MI	Combination	44.5
Solomon 1989	37	USA	consecutive	prospective	7734	MI	Combination	14.5
Mukerji 1989	38	USA	nr	retrospective	250	CHD	Angiography	50.0
Cunningham 1989	39	USA	consecutive	prospective	7734	MI	Combination	14.5
Lee 1989	40	USA	consecutive	prospective	7734	MI	Combination	14.5
Berger 1990	41	CH	consecutive	prospective	278	MI	Combination	36.0
Ananich 1990	42	RUS	nr	prospective	173	MI	Angiography	24.3
Lee 1990	43	USA	consecutive	prospective	7734	MI	Combination	13.6
Weaver 1990	44	USA	nr	prospective	2472	MI	Clinical course	18.3
Hoberg 1990	45	D	consecutive	prospective	220	CHD	Angiography	54.2

Study		Country	Design		Size (n)	Reference disease	Reference standard	Patients with disease (%)
Hartz 1990	46	USA	consecutive	prospective	275	CHD	Angiography	68.4
Ohman 1996	47	IRL	consecutive	prospective	82	MI	Combination	62.0
Jonsbu 1991	48	N	consecutive	prospective	200	MI	Combination	36.5
Gaston-Johansson 199	49	S	consecutive	prospective	94	MI	Combination	40.4
Miranda 1991	50	USA	nr	retrospective	476	CHD	Angiography	75.6
Greene 1991	51	USA	not consecutive	prospective	212	CHD	Angiography	65.1
Elder 1991	52	GB	consecutive	retrospective	268	CHD	Angiography	89.9
Aufderheide 1992	53	USA	nr	prospective	680	MI	Combination	20.7
Ben-Haim 1992	54	USA	nr	prospective	49	MI	Combination	16.3
Herlitz 1992	55	S	consecutive	prospective	4232	MI	Combination	21.8
Barefoot 1992	56	USA	nr	retrospective	1465	CHD	Angiography	68.0
Dilger 1992	57	D	consecutive	prospective	122	MI	Combination	37.0
Lee 1993	58	GB	consecutive	prospective	136	MI	Enzymes	54.4
Hartford 1993	59	S	consecutive	prospective	226	MI	Combination	48.8
Aase 1993	60	N	consecutive	prospective	918	MI	Clinical course	44.4
Hofgren 1994	61	S	consecutive	prospective	889	MI	Combination	49.0
Mayou 1994	62	GB	consecutive	prospective	94	CHD	Clinical course	36.7
Bakker 1994	63	NL	consecutive	prospective	297	MI	Combination	51.6
Lee 1994	64	GB	consecutive	prospective	94	MI	Combination	46.8
Eriksson 1994	65	S	consecutive	prospective	80	MI	Combination	50.0
Chin 1994	66	USA	consecutive	prospective	500	MCE	Clinical course	21.8
Chattington 1994	67	GB	nr	prospective	156	MI	Combination	26.0
Grijseels 1995	68	NL	consecutive	prospective	1005	ACS	Combination	42.0
Herlitz 1995	69	S	consecutive	prospective	503	MI	Combination	21.4
Warner 1995	70	USA	nr	prospective	55	CHD	Combination	40.0
Hofgren 1995	71	S	consecutive	prospective	914	MI	Combination	49.7
Everts 1996	72	S	consecutive	prospective	903	MI	Combination	50.0
Goldman 1996	73	USA	consecutive	prospective	4676	MCE	Combination	3.6
Tighe 1996	74	IRL	consecutive	prospective	264	MI	Combination	32.6
Fraenkel 1996	75	IL	not consecutive	retrospective	114	CHD	Angiography	57.9
Cooke 1997	76	GB	consecutive	prospective	130	CHD	Angiography	50.0
Vikenes 1997	77	N	consecutive	prospective	113	MI	Combination	63.0
Pfister 1997	78	CH	consecutive	prospective	364	MI	Combination	18.3
Kudenchuk 1998	79	USA	consecutive	prospective	2665	ACS	Combination	47.1
Lopez-Jimenez 1998	80	USA	consecutive	prospective	2694	MI	Combination	6.4
Ladwig 1998	81	D	consecutive	prospective	89	CHD	Angiography	64.9
Atwood 1998	82	USA	consecutive	retrospective	1384	CHD	Angiography	59.6
Pope 1998	83	USA	consecutive	prospective	10689	ACS	Combination	23.8
Kontos 1998	84	USA	consecutive	prospective	260	MCE	Clinical course	17.3
Zimmerman 1999	85	USA	consecutive	prospective	955	ACS	Combination	33.7
Holmvang 1999	86	DK	nr	prospective	629	MI	Combination	6.5
Aase 1999	87	N	nr	prospective	493	MI	Clinical course	36.1
Gurevitz 2000	88	IL	consecutive	prospective	135	CHD	Angiography	58.5
Bassan 2000	89	BR	consecutive	prospective	1003	MI	Combination	16.5

Study		Country	Design		Size (n)	Reference disease	Reference standard	Patients with disease (%)
Albarran 2000	90	GB	consecutive	retrospective	267	MI	Combination	44.2
Schannwell 2000	91	D	consecutive	prospective	5000	CHD	Angiography	68.2
Hsu 2000	92	SGP	consecutive	prospective	51	MI	Combination	56.9
Ng SM 2001	93	USA	consecutive	prospective	1285	MI	Combination	5.1
Sharaf 2001	94	USA	consecutive	prospective	323	CHD	Angiography	43.0
McCord 2001	95	USA	consecutive	prospective	1024	MI	Combination	8.0
Funk 2001	96	USA	consecutive	prospective	528	ACS	Combination	40.7
Goodacre 2002	97	GB	consecutive	prospective	893	MI	Combination	3.8
Herlitz 2002a	98	S	consecutive	prospective	1592	MI	Combination	17.9
Derhaschnig 2002	99	A	consecutive	prospective	200	ACS	Combination	51.1
Baxt 2002a	100	USA	nr	prospective	2204	ACS	Clinical course	16.4
Vodopiutz 2002	101	A	nr	nr	92	CHD	Combination	46.7
Herlitz 2002	102	S	consecutive	retrospective	930	ACS	Combination	30.0
Albarran 2002	103	GB	consecutive	prospective	541	MI	Combination	49.2
Baxt 2002	104	USA	nr	prospective	2204	MI	Combination	5.8
Schannwell 2002	105	D	consecutive	prospective	180	CHD	Angiography	57.8
Muscholl 2002	106	D	consecutive	prospective	132	MCE	Clinical course	34.1
De 2002	107	CDN	consecutive	retrospective	187	CHD	Angiography	29.4
Milner 2002	108	USA	nr	prospective	522	ACS	Combination	40.8
Bodi 2002	109	E	consecutive	prospective	385	MI	Enzymes	4.2
Shry 2002	110	USA	nr	nr	223	CHD	Combination	33.2
Svensson 2003	111	S	consecutive	prospective	538	MI	Combination	29.5
McClelland 2003	112	GB	consecutive	prospective	103	MI	Combination	51.5
D'Antono 2003	113	CDN	consecutive	prospective	333	MCE	Clinical course	26.4
Henrikson 2003	114	USA	consecutive	prospective	459	CHD	Clinical course	33.9
Miller 2004	115	USA	consecutive	prospective	2992	MCE	Clinical course	2.8
Ismail 2004	116	PK	consecutive	prospective	388	MI	Combination	50.0
Norgaard 2004	117	DK	nr	prospective	1209	MCE	Clinical course	17.8
Bassan 2004	118	BR	consecutive	prospective	1003	ACS	Combination	47.5
Bulgiba 2005	119	MAL	consecutive	retrospective	887	MI	Combination	27.7
Sanfilippo 2005	120	CDN	nr	prospective	158	CHD	Combination	19.0
Wierzbicki 2005	121	PK	nr	prospective	400	CHD	Angiography	50.0
Farwell 2005	122	USA	consecutive	prospective	600	MI	Combination	50.5
Cosin-Sales 2005	123	GB	consecutive	prospective	643	CHD	Angiography	84.9
Rothenbacher 2005	124	D	nr	prospective	788	CHD	Angiography	39.6
Soiza 2005	125	GB	consecutive	prospective	869	ACS	Clinical course	64.3
Wu 2005	126	GB	consecutive	prospective	405	CHD	Angiography	60.2
Svensson 2005	127	S	consecutive	prospective	538	MI	Combination	31.0
Park 2005	128	D	consecutive	prospective	264	CHD	Angiography	77.3
Diercks 2005	129	USA	nr	prospective	664	CHD	Combination	18.4
Schulze 2006	130	D	nr	retrospective	262	CHD	Clinical course	50.0

Study		Country	Design		Size (n)	Reference disease	Reference standard	Patients with disease (%)
Johnston 2006	131	S	not consecutive	nr	921	ACS	Clinical course	53.2
Johnson 2006	132	USA	consecutive	prospective	673	CHD	Angiography	38.8
Shaw 2006	133	USA	nr	prospective	883	CHD	Angiography	38.1
Christenson 2006	134	CDN	consecutive	prospective	769	ACS	Combination	21.5
Comeau 2006	135	CDN	consecutive	prospective	100	ACS	Clinical course	31.0
Green 2006	136	S	consecutive	prospective	634	ACS	Combination	20.5
Bjork 2006	137	S	consecutive	prospective	634	ACS	Combination	20.5
Gorelik 2006	138	IL	consecutive	prospective	921	ACS	Combination	23.8
Bhiladvala 2006	139	S	consecutive	prospective	356	MI	Enzymes	27.8
Alhashemi 2006	140	KSA	consecutive	prospective	64	MI	Combination	64.0
Pekdemir 2006	141	TR	consecutive	prospective	137	ACS	Combination	37.2
Gellerstedt 2006	142	S	consecutive	prospective	503	MI	Combination	20.9
Steele 2006	143	CDN	consecutive	prospective	270	CHD	Combination	30.7
Han 2007	144	USA	nr	prospective	10126	ACS	Combination	8.3
Jerlock 2007	145	S	consecutive	prospective	294	ACS	Combination	18.0
Han 2007	146	USA	consecutive	prospective	10806	ACS	Clinical course	8.1
Sekhri 2007	147	GB	consecutive	prospective	8762	MCE	Clinical course	6.8
Martin 2007	148	GB	consecutive	prospective	151	MI	MRI	50.0
Lewandoski 2007	149	PL	nr	prospective	551	CHD	Angiography	61.0
Ecollan 2007	150	F	consecutive	prospective	108	MI	Enzymes	51.0
Sanchez 2007	151	E	consecutive	prospective	732	ACS	Combination	27.2
Lozano 2007	152	E	consecutive	prospective	191	ACS	Combination	19.9
Zornitzki 2007	153	IL	consecutive	prospective	243	CHD	Angiography	63.0
Min 2007	154	USA	consecutive	retrospective	1127	CHD	Cardiac CT	38.5
Myint 2007	155	GB	consecutive	prospective	187	ACS	Combination	45.9
Høilund-Carlsen 2007	156	DK	consecutive	prospective	357	CHD	Angiography	35.3
Mad 2007	157	A	consecutive	prospective	295	ACS	Combination	50.0
Gorelik 2007	158	IL	consecutive	prospective	1000	ACS	Combination	22.7
Koz 2008	159	TR	consecutive	prospective	102	CHD	Angiography	44.1
Blich 2008	160	IL	consecutive	retrospective	883	MI	Combination	64.8
Body 2008	161	GB	consecutive	prospective	796	MI	Enzymes	18.6
Cury 2008	162	USA	consecutive	prospective	62	ACS	Combination	21.0
Horinaka 2008	163	J	consecutive	prospective	696	CHD	Angiography	83.3
Jung 2008	164	ROK	not consecutive	retrospective	192	CHD	Angiography	47.4
Kim 2008	165	ROK	nr	prospective	40	CHD	Combination	35.0
Liyan 2008	166	CN	consecutive	prospective	113	ACS	Combination	79.7
Rubinshtein 2008	167	IL	consecutive	prospective	545	CHD	Cardiac CT	16.7
Six 2008	168	NL	consecutive	prospective	120	MCE	Combination	24.2
Kumarathurai 2008	169	DK	consecutive	prospective	273	CHD	Perfusion scintigraphy	44.3
Jo 2009	170	ROK	consecutive	prospective	434	CHD	Angiography	51.8
Onishi 2009	171	J	consecutive	prospective	186	CHD	Combination	23.1
Ueno 2009	172	J	consecutive	prospective	36	ACS	Combination	33.3

ACS: acute coronary syndrome; CHD: coronary heart disease; MI: myocardial infarction; MCE: major cardiac event;
n.r.: not reported;
A: Austria; AUS: Australia; B: Belgium; BR: Brazil; CDN: Canada; CH: Switzerland; CN: People's Republic of China; D: Germany; DK: Denmark; E: Spain; F: France; GB: United Kingdom; IL: Israel; IRL: Ireland; J: Japan;

Study	Country	Design	Size (n)	Reference disease	Reference standard	Patients with disease (%)
KSA: Saudi Arabia;<br>MAL: Malaysia; N: Norway; NL: Netherlands; NZ: New Zealand; PK: Pakistan; PL: Poland; ROK: Republic of Korea; RUS: Russia; S: Sweden; SGP: Singapore; TR: Turkey; USA: United States of America						

Reference list of included studies

1. McConahay DR, McCallister BD, Smith RE. Postexercise electrocardiography: correlations with coronary arteriography and left ventricular hemodynamics. Am.J.Cardiol. 1971;**28**:1-9.

2. Sawe U. Pain in acute myocardial infarction. A study of 137 patients in a coronary care unit. Acta Med.Scand. 1971;**190**:79-81.

3. Sawe U. Early diagnosis of acute myocardial infarction with special reference to the diagnosis of the intermediate coronary syndrome. Previous history and acute symptoms in 921 patients admitted to a CCU. Acta Med.Scand.Suppl 1972;**545**:1-25.

4. Sawe U. Early diagnosis of acute myocardial infarction with special reference to the diagnosis of the intermediate coronary syndrome. The early clinical picture of patients admitted to a CCU. Acta Med.Scand.Suppl 1972;**545**:32-76.

5. Piessens J, Van Mieghem W, Kesteloot H, De Geest H. Diagnostic value of clinical history, exercise testing and atrial pacing in patients with chest pain. Am.J.Cardiol. 1974;**33**:351-6.

6. Detry JMR, Kapita BM, Cosyns J. Diagnostic value of history and maximal exercise electrocardiography in men and women suspected of coronary heart disease. Circulation 1977;**56**:756-61.

7. Schroeder JS, Lamb IH, Hu M. The prehospital course of patients with chest pain. Analysis of the prodromal, symptomatic, decision-making, transportation and emergency room periods. Am.J.Med. 1978;**64**:742-8.

8. Waters DD, Halphen C, Theroux P, David PR, Mizgala HF. Coronary artery disease in young women: clinical and angiographic features and correlation with risk factors. Am.J.Cardiol. 1978;**42**:41-7.

9. Plotnick GD, Greene HL, Carliner NH, Becker LC, Fisher ML. Clinical indicators of left main coronary artery disease in unstable angina. Ann.Intern.Med. 1979;**91**:149-53.

10. Schroeder JS, Lamb IH, Hu M. Prodromal characteristics as indicators of cardiac events in patients hospitalized for chest pain. Clin.Cardiol. 1979;**2**:33-9.

11. Pearson TA, Bulkley BH, Achuff SC, Kwiterovich PO, Gordis L. The association of low levels of HDL cholesterol and arteriographically defined coronary artery disease. Am.J.Epidemiol. 1979;**109**:285-95.

12. Van der Does E., Lubsen J, Pool J. Acute myocardial infarction: an easy diagnosis in general practice? J.R.Coll.Gen.Pract. 1980;**30**:405-9.

13. Grande P, Christiansen C, Pedersen A, Christensen MS. Optimal diagnosis in acute myocardial infarction. A cost-effectiveness study. Circulation 1980;**61**:723-8.

14. Short D. Diagnosis of slight and subacute coronary attacks in the community. Br.Heart J. 1981;**45**:299-310.

15. Holmes DR, Jr., Elveback LR, Frye RL, Kottke BA, Ellefson RD. Association of risk factor variables and coronary artery disease documented with angiography. Circulation 1981;**63**:293-9.

16. Chaitman BR, Bourassa MG, Davis K, Rogers WJ, Tyras DH, Berger R *et al.* Angiographic prevalence of high-risk coronary artery disease in patient subsets (CASS). Circulation 1981;**64**:360-7.

17. Craig IH. Accuracy of hospital diagnosis of myocardial infarction. Aust.N.Z.J.Med. 1982;**12**:14-8.

18. Kambara H, Kawashita K, Yoshida A, Kawai C, Tamaki N, Ishii Y *et al.* Identification of patients with coronary artery disease using a scoring system of coronary risk factors, electrocardiography and myocardial perfusion imaging. Jpn.Circ.J. 1982;**46**:235-44.

19. Hickam DH, Sox HC, Jr., Marton KI, Skeff KM, Chin D. A study of the implicit criteria used in diagnosing chest pain. Med.Decis.Making 1982;**2**:403-14.

20. Beunderman R, Duyvis DJ. Myocardial infarction patients during the prodromal and acute phase: a comparison with patients with a diagnosis of 'noncardiac chest pain'. Psychother.Psychosom. 1983;**40**:129-36.

21. Melin JA, Piret LJ, Vanbutsele RJ, Cosyns J, Rousseau MF, Detry JM. Bayesian analysis of stress thallium scintigraphy for the detection of multivessel coronary disease. Eur.Heart J. 1983;**4**:622-31.

22. Sirnes PA, Overskeid K, Pedersen TR, Bathen J, Drivenes A, Froland GS *et al.* Evolution of infarct size during the early use of nifedipine in patients with acute myocardial infarction: the Norwegian Nifedipine Multicenter Trial. Circulation 1984;**70**:638-44.

23. Lee TH, Cook EF, Weisberg M, Sargent RK, Wilson C, Goldman L. Acute chest pain in the emergency room. Identification and examination of low-risk patients. Arch.Intern.Med. 1985;**145**:65-9.

24. Turi ZG, Rutherford JD, Roberts R, Muller JE, Jaffe AS, Rude RE *et al.* Electrocardiographic, enzymatic and scintigraphic criteria of acute myocardial infarction as determined from study of 726 patients (A MILIS Study). Am.J.Cardiol. 1985;**55**:1463-8.

25. Brown AK, Anderson V, Davies L. A survey of patients selected by general practitioners for home care of suspected myocardial infarction: arrhythmia detection using Holter monitoring. Eur.Heart J. 1985;**6**:13-20.

26. Davies HA, Jones DB, Rhodes J, Newcombe RG. Angina-like esophageal pain: differentiation from cardiac pain by history. J Clin.Gastroenterol. 1985;**7**:477-81.

27. Underwood DA, Proudfit WL, Lim J, MacMillan JP. Symptomatic coronary artery disease in patients aged 21 to 30 years. Am.J.Cardiol. 1985;**55**:631-4.

28. Joswig BC, Glover MU, Nelson DP, Handler JB, Henderson J. Analysis of historical variables, risk factors and the resting electrocardiogram as an aid in the clinical diagnosis of recurrent chest pain. Comput.Biol.Med. 1985;**15**:71-80.

29. Logan RL, Wong F, Barclay J. Symptoms associated with myocardial infarction: are they of diagnostic value? N.Z.Med.J. 1986;**99**:276-8.

30. Tierney WM, Fitzgerald J, McHenry R, Roth BJ, Psaty B, Stump DL *et al*. Physicians' estimates of the probability of myocardial infarction in emergency room patients with chest pain. Med.Decis.Making 1986;**6**:12-7.

31. Toyosaki N, Tsuchiya M, Hashimoto T, Kawasaki K, Shiina A, Toyooka T *et al*. Earlobe crease and coronary heart disease in Japanese. Heart Vessels 1986;**2**:161-5.

32. Kladetzky RG, Santosa H, Schulte H, Spiller P. [Significance of lipids in the diagnosis of coronary heart disease in patient groups and individuals]. Z.Kardiol. 1986;**75**:220-4.

33. Madsen JK, Thomsen BL, Sorensen JN, Kjeldgaard KM, Kromann-Andersen B. Risk factors and prognosis after discharge for patients admitted because of suspected acute myocardial infarction with and without confirmed diagnosis. Am.J.Cardiol. 1987;**59**:1064-70.

34. Hedges JR, Rouan GW, Toltzis R, Goldstein-Wayne B, Stein EA. Use of cardiac enzymes identifies patients with acute myocardial infarction otherwise unrecognized in the emergency department. Ann.Emerg.Med. 1987;**16**:248-52.

35. Miller DH, Kligfield P, Schreiber TL, Borer JS. Relationship of prior myocardial infarction to false-positive electrocardiographic diagnosis of acute injury in patients with chest pain. Arch.Intern.Med. 1987;**147**:257-61.

36. Herlihy T, McIvor ME, Cummings CC, Siu CO, Alikahn M. Nausea and vomiting during acute myocardial infarction and its relation to infarct size and location. Am.J.Cardiol. 1987;**60**:20-2.

37. Solomon CG, Lee TH, Cook EF, Weisberg MC, Brand DA, Rouan GW *et al*. Comparison of clinical presentation of acute myocardial infarction in patients older than 65 years of age to younger patients: the Multicenter Chest Pain Study experience. Am.J.Cardiol. 1989;**63**:772-6.

38. Mukerji V, Alpert MA, Hewett JE, Parker BM. Can patients with chest pain and normal coronary arteries be discriminated from those with coronary artery disease prior to coronary angiography? Angiology 1989;**40**:276-82.

39. Cunningham MA, Lee TH, Cook EF, Brand DA, Rouan GW, Weisberg MC *et al*. The effect of gender on the probability of myocardial infarction among emergency department patients with acute chest pain: a report from the Multicenter Chest Pain Study Group. J.Gen.Intern.Med. 1989;**4**:392-8.

40. Lee TH, Weisberg MC, Brand DA, Rouan GW, Goldman L. Candidates for thrombolysis among emergency room patients with acute chest pain. Potential true- and false-positive rates. Ann.Intern.Med. 1989;**110**:957-62.

41. Berger JP, Buclin T, Haller E, Van MG, Yersin B. Right arm involvement and pain extension can help to differentiate coronary diseases from chest pain of other origin: a prospective emergency ward study of 278 consecutive patients admitted for chest pain. J.Intern.Med. 1990;**227**:165-72.

42. Ananich VA, Gratsianskii NA, Karasev AV, Olferiev AM, Nechaev AS, Yanus VM *et al*. New-onset angina pectoris: initial characteristics and results of a 6 to 12-month follow-up. Cor Vasa 1990;**32**:99-106.

43. Lee TH, Cook EF, Weisberg MC, Rouan GW, Brand DA, Goldman L. Impact of the availability of a prior electrocardiogram on the triage of the patient with acute chest pain. J Gen.Intern Med 1990;**5**:381-8.

44. Weaver WD, Eisenberg MS, Martin JS, Litwin PE, Shaeffer SM, Ho MT *et al*. Myocardial Infarction Triage and Intervention Project--phase I: patient characteristics and feasibility of prehospital initiation of thrombolytic therapy. J.Am.Coll.Cardiol. 1990;**15**:925-31.

45. Hoberg E, Kunze B, Rausch S, Konig J, Schafer H, Kubler W. Diagnostic value of ambulatory Holter monitoring for the detection of coronary artery disease in patients with variable threshold angina pectoris. Am.J.Cardiol. 1990;**65**:1078-83.

46. Hartz A, Grubb B, Wild R, Van Nort JJ, Kuhn E, Freedman D *et al*. The association of waist hip ratio and angiographically determined coronary artery disease. Int.J.Obes. 1990;**14**:657-65.

47. Ohman EM, Casey C, Bengtson JR, Pryor D, Tormey W, Horgan JH. Early detection of acute myocardial infarction: additional diagnostic information from serum concentrations of myoglobin in patients without ST elevation. Br.Heart J. 1990;**63**:335-8.

48. Jonsbu J, Rollag A, Aase O, Lippestad CT, Arnesen KE, Erikssen J *et al*. Rapid and correct diagnosis of myocardial infarction: standardized case history and clinical examination provide important information for correct referral to monitored beds. J.Intern.Med. 1991;**229**:143-9.

49. Gaston-Johansson F, Hofgren C, Watson P, Herlitz J. Myocardial infarction pain: systematic description and analysis. Intensive Care Nurs. 1991;**7**:3-10.

50. Miranda CP, Lehmann KG, Froelicher VF. Correlation between resting ST segment depression, exercise testing, coronary angiography, and long-term prognosis. Am.Heart J. 1991;**122**:1617-28.

51. Greene AF, Schocken DD, Spielberger CD. Self-report of chest pain symptoms and coronary artery disease in patients undergoing angiography. Pain 1991;**47**:319-24.

52. Elder AT, Shaw TR, Turnbull CM, Starkey IR. Elderly and younger patients selected to undergo coronary angiography. BMJ 1991;**303**:950-3.

53. Aufderheide TP, Keelan MH, Hendley GE, Robinson NA, Hastings TE, Lewin RF *et al*. Milwaukee Prehospital Chest Pain Project--phase I: feasibility and accuracy of prehospital thrombolytic candidate selection. Am.J.Cardiol. 1992;**69**:991-6.

54. Ben-Haim SA, Gil A, Edoute Y. Beat-to-beat morphologic variability of the electrocardiogram for the evaluation of chest pain in the emergency room. Am.J.Cardiol. 1992;**70**:1139-42.

55. Herlitz J, Karlson BW, Richter A, Strombom U, Hjalmarson A. Prognosis for patients with initially suspected acute myocardial infarction in relation to presence of chest pain. Clin.Cardiol. 1992;**15**:570-6.

56. Barefoot JC, Beckham JC, Peterson BL, Haney TL, Williams RB, Jr. Measures of neuroticism and disease status in coronary angiography patients. J Consult Clin.Psychol. 1992;**60**:127-32.

57. Dilger J, Pietsch-Breitfeld B, Stein W, Overkamp D, Ickrath O, Renn W *et al*. Simple computer-assisted diagnosis of acute myocardial infarction in patients with acute thoracic pain. Methods Inf.Med. 1992;**31**:263-7.

58. Lee HS, Cross SJ, Rawles JM, Jennings KP. Patients with suspected myocardial infarction who present with ST depression. Lancet 1993;**342**:1204-7.

59. Hartford M, Karlson BW, Sjolin M, Holmberg S, Herlitz J. Symptoms, thoughts, and environmental factors in suspected acute myocardial infarction. Heart Lung 1993;**22**:64-70.

60. Aase O, Jonsbu J, Liestol K, Rollag A, Erikssen J. Decision support by computer analysis of selected case history variables in the emergency room among patients with acute chest pain. Eur.Heart J 1993;**14**:433-40.

61. Hofgren C, Karlson BW, Gaston-Johansson F, Herlitz J. Word descriptors in suspected acute myocardial infarction: a comparison between patients with and without confirmed myocardial infarction. Heart Lung 1994;**23**:397-403.

62. Mayou R, Bryant B, Forfar C, Clark D. Non-cardiac chest pain and benign palpitations in the cardiac clinic. Br.Heart J. 1994;**72**:548-53.

63. Bakker AJ, Gorgels JP, van VB, Koelemay MJ, Smits R, Tijssen JG *et al*. Contribution of creatine kinase MB mass concentration at admission to early diagnosis of acute myocardial infarction. Br.Heart J. 1994;**72**:112-8.

64. Lee HS, Cross SJ, Garthwaite P, Dickie A, Ross I, Walton S *et al*. Comparison of the value of novel rapid measurement of myoglobin, creatine kinase, and creatine kinase-MB with the electrocardiogram for the diagnosis of acute myocardial infarction. Br.Heart J. 1994;**71**:311-5.

65. Eriksson B, Vuorisalo D, Sylven C. Diagnostic potential of chest pain characteristics in coronary care. J.Intern.Med. 1994;**235**:473-8.

66. Chin MH, Cook EF, Lee TH, Goldman L. Correlates of major complications and mortality in patients presenting to the emergency department with chest pain and more than bibasilar rales. J.Gen.Intern.Med. 1994;**9**:659-65.

67. Chattington P, Clarke D, Neithercut WD. Timed sequential analysis of creatine kinase in the diagnosis of myocardial infarction in patients over 65 years of age. J.Clin.Pathol. 1994;**47**:995-8.

68. Grijseels EW, Deckers JW, Hoes AW, Hartman JA, Van der DE, Van LE *et al.* Pre-hospital triage of patients with suspected myocardial infarction. Evaluation of previously developed algorithms and new proposals. Eur.Heart J. 1995;**16**:325-32.

69. Herlitz J, Bang A, Isaksson L, Karlsson T. Ambulance despatchers' estimation of intensity of pain and presence of associated symptoms in relation to outcome in patients who call for an ambulance because of acute chest pain. Eur.Heart J 1995;**16**:1789-94.

70. Warner CD. Somatic awareness and coronary artery disease in women with chest pain. Heart Lung 1995;**24**:436-43.

71. Hofgren C, Karlson BW, Herlitz J. Prodromal symptoms in subsets of patients hospitalized for suspected acute myocardial infarction. Heart Lung 1995;**24**:3-10.

72. Everts B, Karlson BW, Wahrborg P, Hedner T, Herlitz J. Localization of pain in suspected acute myocardial infarction in relation to final diagnosis, age and sex, and site and type of infarction. Heart Lung 1996;**25**:430-7.

73. Goldman L, Cook EF, Johnson PA, Brand DA, Rouan GW, Lee TH. Prediction of the need for intensive care in patients who come to the emergency departments with acute chest pain. N.Engl.J.Med. 1996;**334**:1498-504.

74. Tighe M, Kellett J, Corry R, Reddan E, Ryan B. The early diagnosis of acute myocardial infarction. Comparison of a simple algorithm with a computer program for electrocardiogram interpretation. Ir.J.Med.Sci. 1996;**165**:159-63.

75. Fraenkel YM, Kindler S, Melmed RN. Differences in cognitions during chest pain of patients with panic disorder and ischemic heart disease. Depress.Anxiety. 1996;**4**:217-22.

76. Cooke RA, Smeeton N, Chambers JB. Comparative study of chest pain characteristics in patients with normal and abnormal coronary angiograms. Heart 1997;**78**:142-6.

77. Vikenes K, von der LG, Farstad M, Nordrehaug JE. Clinical applicability of creatine kinase MB mass and the electrocardiogram versus conventional cardiac enzymes in the diagnosis of acute myocardial infarction. Int.J.Cardiol. 1997;**59**:11-20.

78. Pfister R, Gaillet R, Saner H, Pirovino M, Castelli I, Gaspoz JM. Die Vorhospitalphase von Patienten mit Verdacht auf akuten Myokardinfarkt: Ergebnisse der "Oltner Herznotfallstudie" [The prehospital phase of patients with suspected acute myocardial infarct: results of the Oltner Cardiac Emergency Study]. Schweiz.Med.Wochenschr. 1997;**127**:479-88.

79. Kudenchuk PJ, Maynard C, Cobb LA, Wirkus M, Martin JS, Kennedy JW *et al.* Utility of the prehospital electrocardiogram in diagnosing acute coronary syndromes: the Myocardial Infarction Triage and Intervention (MITI) Project. J.Am.Coll.Cardiol. 1998;**32**:17-27.

80. Lopez-Jimenez F, Goldman L, Johnson PA, Polanczyk CA, Cook EF, Fleischmann KE *et al*. Effect of diabetes mellitus on the presentation and triage of patients with acute chest pain without known coronary artery disease. Am.J.Med. 1998;**105**:500-5.

81. Ladwig KH, Hoberg E, Busch R. [Psychological comorbidity in patients with alarming chest pain symptoms]. Psychother.Psychosom.Med Psychol. 1998;**48**:46-54.

82. Atwood JE, Do D, Froelicher V, Chilton R, Dennis C, Froning J *et al*. Can computerization of the exercise test replace the cardiologist? Am.Heart J. 1998;**136**:543-52.

83. Pope JH, Ruthazer R, Beshansky JR, Griffith JL, Selker HP. Clinical Features of Emergency Department Patients Presenting with Symptoms Suggestive of Acute Cardiac Ischemia: A Multicenter Study. J.Thromb.Thrombolysis. 1998;**6**:63-74.

84. Kontos MC, Arrowood JA, Paulsen WH, Nixon JV. Early echocardiography can predict cardiac events in emergency department patients with chest pain. Ann.Emerg.Med. 1998;**31**:550-7.

85. Zimmerman J, Fromm R, Meyer D, Boudreaux A, Wun CC, Smalling R *et al*. Diagnostic marker cooperative study for the diagnosis of myocardial infarction. Circulation 1999;**99**:1671-7.

86. Holmvang L, Clemmensen P, Wagner G, Grande P. Admission standard electrocardiogram for early risk stratification in patients with unstable coronary artery disease not eligible for acute revascularization therapy: a TRIM substudy. ThRombin Inhibition in Myocardial Infarction. Am.Heart J. 1999;**137**:24-33.

87. Aase O. Clinical experience with a decision support computer program using Bayes' theorem to diagnose chest pain patients. Cardiology 1999;**92**:128-34.

88. Gurevitz O, Jonas M, Boyko V, Rabinowitz B, Reicher-Reiss H. Clinical profile and long-term prognosis of women < or = 50 years of age referred for coronary angiography for evaluation of chest pain. Am.J.Cardiol. 2000;**85**:806-9.

89. Bassan R, Scofano M, Gamarski R, Dohmann HF, Pimenta L, Volschan A *et al*. Chest pain in the emergency room. Importance of a systematic approach. Arq Bras.Cardiol. 2000;**74**:13-29.

90. Albarran JW, Durham B, Chappel G, Dwight J, Gowers J. Are manual gestures, verbal descriptors and pain radiation as reported by patients reliable indicators of myocardial infarction? Preliminary findings and implications. Intensive Crit Care Nurs. 2000;**16**:98-110.

91. Schannwell CM, Schoebel FC, Lazica D, Marx R, Plehn G, Leschke M *et al*. [Differences in the clinical performance and initial diagnosis in women with suspected coronary artery disease]. Dtsch.Med.Wochenschr. 2000;**125**:1417-23.

92. Hsu LF, Koh TH, Lim YL. Cardiac marker point-of-care testing: evaluation of rapid on-site biochemical marker analysis for diagnosis of acute myocardial infarction. Ann.Acad.Med.Singapore 2000;**29**:421-7.

93. Ng SM, Krishnaswamy P, Morissey R, Clopton P, Fitzgerald R, Maisel AS. Ninety-minute accelerated critical pathway for chest pain evaluation. Am.J.Cardiol. 2001;**88**:611-7.

94. Sharaf BL, Pepine CJ, Kerensky RA, Reis SE, Reichek N, Rogers WJ *et al*. Detailed angiographic analysis of women with suspected ischemic chest pain (pilot phase data from the NHLBI-sponsored Women's Ischemia Syndrome Evaluation [WISE] Study Angiographic Core Laboratory). Am.J.Cardiol. 2001;**87**:937-41.

95. McCord J, Nowak RM, McCullough PA, Foreback C, Borzak S, Tokarski G *et al*. Ninety-minute exclusion of acute myocardial infarction by use of quantitative point-of-care testing of myoglobin and troponin I. Circulation 2001;**104**:1483-8.

96. Funk M, Naum JB, Milner KA, Chyun D. Presentation and symptom predictors of coronary heart disease in patients with and without diabetes. Am.J.Emerg.Med. 2001;**19**:482-7.

97. Goodacre S, Locker T, Morris F, Campbell S. How useful are clinical features in the diagnosis of acute, undifferentiated chest pain? Acad.Emerg.Med. 2002;**9**:203-8.

98. Herlitz J, Karlson BW, Karlsson T, Svensson L, och Bjorn Kalin EZ. A description of the characteristics and outcome of patients hospitalized for acute chest pain in relation to whether they were admitted to the coronary care unit or not in the thrombolytic era. Int.J.Cardiol. 2002;**82**:279-87.

99. Derhaschnig U, Laggner AN, Roggla M, Hirschl MM, Kapiotis S, Marsik C *et al*. Evaluation of coagulation markers for early diagnosis of acute coronary syndromes in the emergency room. Clin.Chem. 2002;**48**:1924-30.

100. Baxt WG, Shofer FS, Sites FD, Hollander JE. A neural network aid for the early diagnosis of cardiac ischemia in patients presenting to the emergency department with chest pain. Ann Emerg Med 2002;**40**:575-83.

101. Vodopiutz J, Poller S, Schneider B, Lalouschek J, Menz F, Stollberger C. Chest pain in hospitalized patients: cause-specific and gender-specific differences. J Womens Health (Larchmt.) 2002;**11**:719-27.

102. Herlitz J, Starke M, Hansson E, Ringvall E, Karlson BW, Waagstein L. Early identification of patients with an acute coronary syndrome as assessed by dispatchers and the ambulance crew. Am.J.Emerg.Med. 2002;**20**:196-201.

103. Albarran J, Durham B, Gowers J, Dwight J, Chappell G. Is the radiation of chest pain a useful indicator of myocardial infarction? A prospective study of 541 patients. Accid.Emerg.Nurs. 2002;**10**:2-9.

104. Baxt WG, Shofer FS, Sites FD, Hollander JE. A neural computational aid to the diagnosis of acute myocardial infarction. Ann.Emerg.Med. 2002;**39**:366-73.

105. Schannwell CM, Lazica D, Plehn G, Leschke M, Strauer BE. [The current status of noninvasive cardiac diagnosis in women with suspected coronary heart disease]. Med.Klin.(Munich) 2002;**97**:513-21.

106. Muscholl MW, Oswald M, Mayer C, von Scheidt W. Prognostic value of 2D echocardiography in patients presenting with acute chest pain and non-diagnostic ECG for ST-elevation myocardial infarction. Int.J Cardiol. 2002;**84**:217-25.

107. De S, Searles G, Haddad H. The prevalence of cardiac risk factors in women 45 years of age or younger undergoing angiography for evaluation of undiagnosed chest pain. Can.J.Cardiol. 2002;**18**:945-8.

108. Milner KA, Funk M, Arnold A, Vaccarino V. Typical symptoms are predictive of acute coronary syndromes in women. Am.Heart J 2002;**143**:283-8.

109. Bodi V, Facila L, Sanchis J, Llacer A, Nunez J, Mainar L *et al*. [Short-term prognosis of patients admitted for probable acute coronary syndrome without ST-segment elevation. Role of new myocardial damage markers and acute-phase reactants]. Rev.Esp.Cardiol. 2002;**55**:823-30.

110. Shry EA, Dacus J, Van De GE, Hjelkrem M, Stajduhar KC, Steinhubl SR. Usefulness of the response to sublingual nitroglycerin as a predictor of ischemic chest pain in the emergency department. Am.J.Cardiol. 2002;**90**:1264-6.

111. Svensson L, Isaksson L, Axelsson C, Nordlander R, Herlitz J. Predictors of myocardial damage prior to hospital admission among patients with acute chest pain or other symptoms raising a suspicion of acute coronary syndrome. Coron.Artery Dis. 2003;**14**:225-31.

112. McClelland AJ, Owens CG, Menown IB, Lown M, Adgey AA. Comparison of the 80-lead body surface map to physician and to 12-lead electrocardiogram in detection of acute myocardial infarction. Am.J.Cardiol. 2003;**92**:252-7.

113. D'Antono B, Dupuis G, Fleet R, Marchand A, Burelle D. Sex differences in chest pain and prediction of exercise-induced ischemia. Can.J.Cardiol. 2003;**19**:515-22.

114. Henrikson CA, Howell EE, Bush DE, Miles JS, Meininger GR, Friedlander T *et al*. Chest pain relief by nitroglycerin does not predict active coronary artery disease. Ann.Intern.Med. 2003;**139**:979-86.

115. Miller CD, Lindsell CJ, Khandelwal S, Chandra A, Pollack CV, Tiffany BR *et al*. Is the initial diagnostic impression of "noncardiac chest pain" adequate to exclude cardiac disease? Ann.Emerg.Med. 2004;**44**:565-74.

116. Ismail J, Jafar TH, Jafary FH, White F, Faruqui AM, Chaturvedi N. Risk factors for non-fatal myocardial infarction in young South Asian adults. Heart 2004;**90**:259-63.

117. Norgaard BL, Andersen K, Thygesen K, Ravkilde J, Abrahamsson P, Grip L *et al*. Long term risk stratification of patients with acute coronary syndromes: characteristics of troponin T testing and continuous ST segment monitoring. Heart 2004;**90**:739-44.

118. Bassan R, Pimenta L, Scofano M, Soares JF. Accuracy of a Neural Diagnostic Tree for the Identification of Acute Coronary Syndrome in Patients With Chest Pain and No ST-Segment Elevation. Crit Pathw.Cardiol. 2004;**3**:72-8.

119. Bulgiba AM, Razaz M. How well can signs and symptoms predict AMI in the Malaysian population? Int.J Cardiol. 2005;**102**:87-93.

120. Sanfilippo AJ, Abdollah H, Knott C, Link C, Hopman W. Defining low risk for coronary heart disease among women with chest pain syndrome: a prospective evaluation. J Womens Health (Larchmt.) 2005;**14**:240-7.

121. Wierzbicki AS, Nishtar S, Lumb PJ, Lambert-Hammill M, Turner CN, Crook MA *et al*. Metabolic syndrome and risk of coronary heart disease in a Pakistani cohort. Heart 2005;**91**:1003-7.

122. Farwell WR, Sesso HD, Buring JE, Gaziano JM. Non-high-density lipoprotein cholesterol versus low-density lipoprotein cholesterol as a risk factor for a first nonfatal myocardial infarction. Am.J.Cardiol. 2005;**96**:1129-34.

123. Cosin-Sales J, Kaski JC, Christiansen M, Kaminski P, Oxvig C, Overgaard MT *et al*. Relationship among pregnancy associated plasma protein-A levels, clinical characteristics, and coronary artery disease extent in patients with chronic stable angina pectoris. Eur.Heart J. 2005;**26**:2093-8.

124. Rothenbacher D, Brenner H, Marz W, Koenig W. Adiponectin, risk of coronary heart disease and correlations with cardiovascular risk markers. Eur.Heart J. 2005;**26**:1640-6.

125. Soiza RL, Leslie SJ, Harrild K, Peden NR, Hargreaves AD. Age-dependent differences in presentation, risk factor profile, and outcome of suspected acute coronary syndrome. J.Am.Geriatr.Soc. 2005;**53**:1961-5.

126. Wu EB, Hodson F, Chambers JB. A simple score for predicting coronary artery disease in patients with chest pain. QJM. 2005;**98**:803-11.

127. Svensson L, Axelsson C, Nordlander R, Herlitz J. Prehospital identification of acute coronary syndrome/myocardial infarction in relation to ST elevation. Int.J Cardiol. 2005;**98**:237-44.

128. Park JW, Hill PM, Chung N, Hugenholtz PG, Jung F. Magnetocardiography predicts coronary artery disease in patients with acute chest pain. Ann.Noninvasive.Electrocardiol. 2005;**10**:312-23.

129. Diercks DB, Boghos E, Guzman H, Amsterdam EA, Kirk JD. Changes in the numeric descriptive scale for pain after sublingual nitroglycerin do not predict cardiac etiology of chest pain. Ann.Emerg.Med. 2005;**45**:581-5.

130. Schulze F, Lenzen H, Hanefeld C, Bartling A, Osterziel KJ, Goudeva L *et al*. Asymmetric dimethylarginine is an independent risk factor for coronary heart disease: results from the multicenter Coronary Artery Risk Determination investigating the Influence of ADMA Concentration (CARDIAC) study. Am.Heart J. 2006;**152**:493-8.

131. Johnston N, Jernberg T, Lagerqvist B, Siegbahn A, Wallentin L. Improved identification of patients with coronary artery disease by the use of new lipid and lipoprotein biomarkers. Am.J.Cardiol. 2006;**97**:640-5.

132. Johnson BD, Shaw LJ, Pepine CJ, Reis SE, Kelsey SF, Sopko G *et al*. Persistent chest pain predicts cardiovascular events in women without obstructive coronary artery disease: results from the NIH-NHLBI-sponsored Women's Ischaemia Syndrome Evaluation (WISE) study. Eur.Heart J. 2006;**27**:1408-15.

133. Shaw LJ, Merz CN, Pepine CJ, Reis SE, Bittner V, Kip KE *et al*. The economic burden of angina in women with suspected ischemic heart disease: results from the National Institutes of Health--National Heart, Lung, and Blood Institute--sponsored Women's Ischemia Syndrome Evaluation. Circulation 2006;**114**:894-904.

134. Christenson J, Innes G, McKnight D, Thompson CR, Wong H, Yu E *et al*. A clinical prediction rule for early discharge of patients with chest pain. Ann.Emerg.Med. 2006;**47**:1-10.

135. Comeau A, Jensen L, Burton JR. Can symptom presentation predict unstable angina/non-ST-segment elevation myocardial infarction in a moderate-risk cohort? Eur.J.Cardiovasc.Nurs. 2006;**5**:127-36.

136. Green M, Bjork J, Forberg J, Ekelund U, Edenbrandt L, Ohlsson M. Comparison between neural networks and multiple logistic regression to predict acute coronary syndrome in the emergency room. Artif.Intell.Med. 2006;**38**:305-18.

137. Bjork J, Forberg JL, Ohlsson M, Edenbrandt L, Ohlin H, Ekelund U. A simple statistical model for prediction of acute coronary syndrome in chest pain patients in the emergency department. BMC.Med.Inform.Decis.Mak. 2006;**6**:28.

138. Gorelik O, moznino-Sarafian D, Yarovoi I, Alon I, Shteinshnaider M, Chachashvily S *et al*. Patient-related variables predicting acute coronary syndrome following admission for chest pain of possible coronary origin. Coron.Artery Dis. 2006;**17**:15-21.

139. Bhiladvala P, Strandberg K, Stenflo J, Holm J. Early identification of acute myocardial infarction by activated protein C--protein C inhibitor complex. Thromb.Res. 2006;**118**:213-9.

140. Alhashemi JA. Diagnostic accuracy of a bedside qualitative immunochromatographic test for acute myocardial infarction. Am J Emerg.Med 2006;**24**:149-55.

141. Pekdemir M, Karaca I, Cevik Y, Yanturali S, Ilkay E. The diagnostic value of QT dispersion for acute coronary syndrome in patients presenting with chest pain and nondiagnostic initial electrocardiograms. Mt.Sinai J.Med. 2006;**73**:813-7.

142. Gellerstedt M, Bang A, Herlitz J. Could a computer-based system including a prevalence function support emergency medical systems and improve the allocation of life support level? Eur.J.Emerg.Med. 2006;**13**:290-4.

143. Steele R, McNaughton T, McConahy M, Lam J. Chest pain in emergency department patients: if the pain is relieved by nitroglycerin, is it more likely to be cardiac chest pain? CJEM. 2006;**8**:164-9.

144. Han JH, Lindsell CJ, Hornung RW, Lewis T, Storrow AB, Hoekstra JW *et al*. The elder patient with suspected acute coronary syndromes in the emergency department. Acad.Emerg.Med. 2007;**14**:732-9.

145. Jerlock M, Welin C, Rosengren A, Gaston-Johansson F. Pain characteristics in patients with unexplained chest pain and patients with ischemic heart disease. Eur.J.Cardiovasc.Nurs. 2007;**6**:130-6.

146. Han JH, Lindsell CJ, Storrow AB, Luber S, Hoekstra JW, Hollander JE *et al*. The role of cardiac risk factor burden in diagnosing acute coronary syndromes in the emergency department setting. Ann.Emerg.Med. 2007;**49**:145-52, 152.

147. Sekhri N, Feder GS, Junghans C, Hemingway H, Timmis AD. How effective are rapid access chest pain clinics? Prognosis of incident angina and non-cardiac chest pain in 8762 consecutive patients. Heart 2007;**93**:458-63.

148. Martin TN, Groenning BA, Murray HM, Steedman T, Foster JE, Elliot AT *et al*. ST-segment deviation analysis of the admission 12-lead electrocardiogram as an aid to early diagnosis of acute myocardial infarction with a cardiac magnetic resonance imaging gold standard. J.Am.Coll.Cardiol. 2007;**50**:1021-8.

149. Lewandowski M, Szwed H, Kowalik I. Searching for the optimal strategy for the diagnosis of stable coronary artery disease. Cost-effectiveness of the new algorithm. Cardiology Journal 2007;**14**:544-51.

150. Ecollan P, Collet JP, Boon G, Tanguy ML, Fievet ML, Haas R *et al*. Pre-hospital detection of acute myocardial infarction with ultra-rapid human fatty acid-binding protein (H-FABP) immunoassay. Int.J Cardiol. 2007;**119**:349-54.

151. Sanchez M, Lopez B, Bragulat E, Gomez-Angelats E, Jimenez S, Ortega M *et al*. Triage flowchart to rule out acute coronary syndrome. Am.J.Emerg.Med. 2007;**25**:865-72.

152. Lozano T, Ena J, Almenar V, Graells M, Molina J, Antorrena I *et al*. [Evaluation of patients with acute chest pain of uncertain origin by means of serial measurement of high-sensitivity C-reactive protein]. Rev.Esp.Cardiol. 2007;**60**:817-24.

153. Zornitzki T, Ayzenberg O, Gandelman G, Vered S, Yaskil E, Faraggi D *et al*. Diabetes, but not the metabolic syndrome, predicts the severity and extent of coronary artery disease in women. QJM. 2007;**100**:575-81.

154. Min JK, Shaw LJ, Devereux RB, Okin PM, Weinsaft JW, Russo DJ *et al*. Prognostic value of multidetector coronary computed tomographic angiography for prediction of all-cause mortality. J.Am.Coll.Cardiol. 2007;**50**:1161-70.

155. Myint PK, Al Jawad M, Chacko SM, Chu GS, Vowler SL, May HM. Prevalence, characteristics and outcomes of people aged 65 years and over with an incidental rise in cardiac troponin I. An observational prospective cohort study. Cardiology 2008;**110**:62-7.

156. Høilund-Carlsen PF, Johansen A, Vach W, Christensen HW, Møldrup M, Haghfelt T. High probability of disease in angina pectoris patients: Is clinical estimation reliable? Can.J.Cardiol. 2007;**23**:641-7.

157. Mad P, Domanovits H, Fazelnia C, Stiassny K, Russmuller G, Cseh A *et al*. Human heart-type fatty-acid-binding protein as a point-of-care test in the early diagnosis of acute myocardial infarction. QJM. 2007;**100**:203-10.

158. Gorelik O, Almoznino-Sarafian D, Yarovoi I, Alon I, Shteinshnaider M, Tzur I *et al*. Patient-dependent variables affecting treatment and prediction of acute coronary syndrome are age-related. A study performed in Israel. Int.J.Cardiol. 2007;**121**:163-70.

159. Koz C, Baysan O, Hasimi A, Cihan M, Uzun M, Yokusoglu M *et al*. Conventional and non-conventional coronary risk factors in male premature coronary artery disease patients already having a low Framingham risk score. Acta Cardiol. 2008;**63**:623-8.

160. Blich M, Sebbag A, Attias J, Aronson D, Markiewicz W. Cardiac troponin I elevation in hospitalized patients without acute coronary syndromes. Am.J.Cardiol. 2008;**101**:1384-8.

161. Body R, McDowell G, Carley S, Mackway-Jones K. Do risk factors for chronic coronary heart disease help diagnose acute myocardial infarction in the Emergency Department? Resuscitation 2008;**79**:41-5.

162. Cury RC, Shash K, Nagurney JT, Rosito G, Shapiro MD, Nomura CH *et al*. Cardiac magnetic resonance with T2-weighted imaging improves detection of patients with acute coronary syndrome in the emergency department. Circulation 2008;**118**:837-44.

163. Horinaka S, Yabe A, Yagi H, Ishimura K, Hara H, Iemua T *et al*. Comparison of Atherosclerotic Indicators Between Cardio Ankle Vascular Index and Brachial Ankle Pulse Wave Velocity. Angiology 2008.

164. Jung CH, Rhee EJ, Shin HS, Jo SK, Won JC, Park CY *et al*. Higher serum free thyroxine levels are associated with coronary artery disease. Endocr.J. 2008;**55**:819-26.

165. Kim JH, Choi JH, Lee HK, Bae WH, Chun KJ, Kim YS *et al*. Ischemia-modified albumin (IMA) is not useful for detecting myocardial ischemia during symptom-limited exercise stress tests. Korean J.Intern.Med. 2008;**23**:121-6.

166. Liyan C, Jie Z, Yonghua W, Xiaozhou H. Assay of ischemia-modified albumin and C-reactive protein for early diagnosis of acute coronary syndromes. J.Clin.Lab Anal. 2008;**22**:45-9.

167. Rubinshtein R, Halon DA, Gaspar T, Peled N, Lewis BS. Cardiac computed tomographic angiography for risk stratification and prediction of late cardiovascular outcome events in patients with a chest pain syndrome. Int.J.Cardiol. 2008.

168. Six AJ, Backus BE, Kelder JC. Chest pain in the emergency room: value of the HEART score. Neth.Heart J. 2008;**16**:191-6.

169. Kumarathurai P, Farooq MK, Christensen HW, Vach W, Hoilund-Carlsen PF. Muscular tenderness in the anterior chest wall in patients with stable angina pectoris is associated with normal myocardial perfusion. J.Manipulative Physiol Ther. 2008;**31**:344-7.

170. Jo SH, Kim SG, Choi YJ, Joo NR, Cho GY, Choi SR *et al*. KLOTHO gene polymorphism is associated with coronary artery stenosis but not with coronary calcification in a Korean population. Int.Heart J. 2009;**50**:23-32.

171. Onishi T, Uematsu M, Nanto S, Morozumi T, Watanabe T, Awata M *et al*. Detection of diastolic abnormality by dyssynchrony imaging: correlation with coronary artery disease in patients presenting with visibly normal wall motion. Circ.J. 2009;**73**:125-31.

172. Ueno K, Anzai T, Jinzaki M, Yamada M, Kohno T, Kawamura A *et al*. Diagnostic capacity of 64-slice multidetector computed tomography for acute coronary syndrome in patients presenting with acute chest pain. Cardiology 2009;**112**:211-8.
