# Supplementary material for: Does the patient with chest pain have a coronary heart disease? Diagnostic value of single symptoms and signs – a meta-analysis
Source: Croat Med J. 2012 Oct;53(5):432–41. doi: 10.3325/cmj.2012.53.432 (PMC3490454; doi:10.3325/cmj.2012.53.432)
Supplement: Supplementary Table 4 [file CroatMedJ_53_s004.pdf]

Supplemental table 4: Degree of heterogeneity assessed quantitative (I^2) and qualitative (visual examination).

Index test	Studies (n)	I^2 Sensitivity (%)	I^2 Specificity (%)	visual examination Forest plot Sensitivity	visual examination Forest plot Specificity
Male sex	102	92.1	97.5	+	++
Higher age	32	98.5	99.5	++	++
History of diabetes mellitus	72	93.5	94.1	+	+
History of dyslipidaemia	46	96.5	98.4	++	++
History of hypertension	70	95.8	97.0	++	++
History of CHD	65	93.8	97.4	+	++
History of MI	52	93.9	96.6	++	++
History of AP	22	97.5	98.7	++	++
Family history of MI	34	92.7	97.3	+	++
Smoking	68	97.3	96.4	++	++
Obesity	12	92.2	95.1	++	++
Menopause	5	0.0	6.6	-	-
Central chest pain	14	98.4	98.0	++	++
Left-sided chest pain	12	96.0	95.2	++	++
Right-sided chest pain	5	93.9	95.1	++	++
Radiation to left arm/ shoulder	12	88.0	91.6	+	+
Radiation to right arm/ shoulder	9	92.5	92.7	+	++
Radiation to back	5	86.1	72.7	+	+
Visceral pain	17	96.2	92.2	+	+
Stabbing pain	11	74.3	96.8	-	+
Burning pain	7	69.8	70.0	-	-
Frightening pain	4	82.4	94.0	+	++
Time since onset of pain > 6 hours	9	95.3	99.1	++	++
Typical angina	14	98.5	99.1	++	++
Atypical angina	5	89.4	99.5	+	++
Pain relief by nitro-glycerine	9	93.6	96.5	+	+
Crescendo angina	3	86.5	92.7	+	++
Pain related to breathing	3	89.3	96.0	+	++
Pain related to effort	8	95.4	93.2	++	++
Sweating	11	97.3	98.0	++	++
Dyspnoea	20	98.4	99.0	++	++
Nausea/ vomiting	13	92.4	90.6	+	++
Dizziness	7	95.0	98.0	+	++
Collapse	9	96.7	97.6	+	++
Palpitations	10	97.2	98.3	++	++
Weakness	6	94.0	93.0	-	+
Fear/ anxiety	3	94.2	52.8	+	-
High blood pressure	3	96.6	97.6	++	++
Tachycardia	3	96.1	97.1	++	++
Bradycardia	3	91.2	34.7	+	-
Rales	8	81.8	98.6	+	++
Pain reproducible by palpation	8	98.0	99.0	+	++
++ very high level of heterogeneity					

+	high level of heterogeneity
-	medium level of heterogeneity

CHD: coronary heart disease; MI: myocardial infarction; AP: angina pectoris
