# Supplementary material for: Does the patient with chest pain have a coronary heart disease? Diagnostic value of single symptoms and signs – a meta-analysis
Source: Croat Med J. 2012 Oct;53(5):432–41. doi: 10.3325/cmj.2012.53.432 (PMC3490454; doi:10.3325/cmj.2012.53.432)
Supplement: Supplementary Table 5 [file CroatMedJ_53_s005.pdf]

Supplemental table 5: Correlation between sensitivity and specificity

Assessed quantitative (Spearman correlation coefficient between logit sensitivity and logit specificity and its *p*-value) and qualitative (visual Examination of Forest plots of sensitivity/ specificity and ROC planes)

Index test	Studies (n)	Spearman	p	R^2	Visual Examination*
Male sex	102	-0.46	<0.001	0.21	-1
Higher age	32	-0.87	<0.001	0.76	-1
History of diabetes mellitus	72	-0.59	<0.001	0.35	-1
History of dyslipidimia	46	-0.64	<0.001	0.41	-1
History of hypertension	70	-0.68	<0.001	0.46	-1
History of CHD	65	-0.21	0.06	0.04	0- (-1)
History of MI	52	-0.19	0.19	0.04	0
History of AP	22	-0.47	0.03	0.22	0-(-1)
Family history of MI	34	-0.72	<0.001	0.52	-1
Smoking	68	-0.83	<0.001	0.69	-1
Obesity	12	-0.69	0.01	0.48	-1
Postmeno-pausal	5	-0.04	0.90	0.00	0
Central chest pain	14	-0.87	<0.001	0.76	-1
Left-sided chest pain	12	-0.75	0.01	0.56	-1
Right-sided chest pain	5	-0.60	0.29	0.36	-1
Radiation to left arm/ shoulder	12	-0.61	0.04	0.37	-1
Radiation to right arm/ shoulder	9	-0.73	0.03	0.53	-1
Radiation to back	5	-0.50	0.39	0.25	-1
Visceral pain	17	-0.56	0.02	0.31	-1
Stabbing pain	11	-0.36	0.27	0.13	0
Burning pain	7	-0.36	0.43	0.13	-1
Frightening pain	4	-0.60	0.40	0.36	-1
Time since onset of pain > 6 hours	9	-0.17	0.67	0.03	0
Typical angina	15	0.09	0.76	0.01	0
Atypical angina	5	-0.70	0.19	0.49	-1
Pain relief by nitro-glycerine	9	-0.75	0.56	0.02	-1
Crescendo angina	3	-0.50	0.67	0.25	-1
Pain related to breathing	3	-1.00	<0.001	1.00	-1
Pain related to effort	8	-0.21	0.61	0.04	-1
Sweating	11	-0.80	<0.01	0.64	-1
Dyspnoea	20	-0.91	<0.001	0.83	-1
Nausea/ vomiting	13	-0.84	<0.001	0.71	-1
Dizziness	7	-0.89	0.01	0.79	-1
Collapse	9	-0.83	0.01	0.69	0-(-1)
Palpitations	10	-0.90	<0.001	0.81	-1
Weakness	6	-0.60	0.21	0.36	-1
Fear/ anxiety	3	-0.50	0.67	0.25	-1
High blood pressure	3	-0.50	0.67	0.25	-1
Tachycardia	3	0.50	0.67	0.25	1
Bradycardia	3	0.50	0.67	0.25	1

Rales	8	-0.57	0.14	0.32	-1
Pain reproducable by palpation	8	-0.95	<0.001	0.90	-1

1: positive correlation between sensitivity and specificity, 0: no correlation, -1: negative correlation
CHD: coronary heart disease; MI: myocardial infarction; AP: angina pectoris
