# Supplementary material for: Does the patient with chest pain have a coronary heart disease? Diagnostic value of single symptoms and signs – a meta-analysis
Source: Croat Med J. 2012 Oct;53(5):432–41. doi: 10.3325/cmj.2012.53.432 (PMC3490454; doi:10.3325/cmj.2012.53.432)
Supplement: Supplementary Table 6 [file CroatMedJ_53_s006.pdf]

Supplemental table 6: Impact of covariates. Table presents results of the meta-regression (p-values) and the visual examination of the ROC planes.

	Male sex	Higher age	History of diabetes mellitus	History of dyslipidaemia	History of hypertension	History of CHD	History of MI	History of AP	Family history of MI	Smoking	Obesity	Central chest pain	Left-sided chest pain	Radiation to left arm/ shoulder	Visceral pain	Stabbing pain	Typical angina	Sweating	Dyspnoea	Nausea/ vomiting	Palpitations
Representative spectrum?	**0.02** -	0.98 -	0.01 -	**0.10** -	0.42	0.32 -	**0.08** +	0.44 +	0.80 -	0.12 -	0.80 -	0.96 -	0.92 -	**0.02** +	0.78 +	0.36 -	0.62 -	0.78 -	0.17 +	0.79 -	0.59 -
Acceptable reference standard?	0.87 +	0.97 -	0.97 -	0.99 -	0.17 +	0.32 -	0.81 -	1.00 -	0.87 -	0.62 -	0.94 -	0.21 +	0.97 -/+	0.97 -	0.72 -	0.62 +	Cons.	0.62 +	0.75 -	0.92 -	1.00 +
Partial verification avoided?	0.90 +	0.52 -	0.97 -	0.18 +	1.00 +	0.50 -/+	0.65 +	0.63 +	Cons.	0.37 -	0.86 -	0.74 -	**0.08** +	0.97 -	0.37 -/+	Cons	Cons.	0.74 -	0.94 -	0.85 -	0.59 -
Differential verification avoided?	0.74 +	0.80 -	0.77 -	0.68 -	0.14 -	1.00 -	0.90 -	0.38 -	**0.06** +	0.84 -	**0.10** +	0.43 +	0.90 -	0.97 -	**0.06** **+/++**	0.22 +/++	0.77 +	0.82 -	0.96 -	0.86 -	0.99 -
Incorporation avoided?	0.55 +	0.32 -	0.94 -	0.97 +	0.34 +	0.29 -	0.82 -	0.88 +	0.64 -/+	0.96 -	0.17 -	0.56 +	0.44 -	0.65 -	0.70 -	0.75 -	Cons.	0.42 -	1.00 -	0.49 +	1.00 -
Details execution index test?	0.30 +	cons.	0.96 -	0.39 +	0.67 -	0.99 -	0.84 -	0.63 -	0.89 -	0.98 -	0.16 +	0.56 +	0.78 -	0.97	0.26 -	0.92 -	1.00 -	0.78 -	0.67 -	0.92 -	0.52 +
Index test results blinded?	0.20 -	0.99 -	0.63 -	0.59 -	0.24 -	0.78 -	0.67 -	0.59 -	0.90 -	0.99 -	0.78 -	0.30 +	0.98 -	0.57 -	0.52 -	0.97 -	1.00 -	0.75 -	0.80 -	0.69 +	0.71 -
Reference standard results blinded?	**<0.01**	0.93	0.68	0.98	0.95	0.23	0.78	Cons.	0.32	0.87	0.89	Cons.	Cons.	0.63	Cons.	0.47	0.72	Cons.	0.83	Cons.	Cons.

	+	-	-	-	+	-	+		-	-	-			+		-	-		-		
Withdrawals explained?	0.96<br>+	0.96<br>-	0.91<br>-	0.99<br>-	0.75<br>-	0.64<br>-	0.65<br>-	0.90<br>-	0.95<br>-	0.95<br>-	**< 0.001**<br>+	0.82<br>-	0.79<br>-	0.79<br>-	0.99<br>-	0.44<br>-	0.69<br>+	0.44<br>-	1.00<br>-	0.78<br>-	0.86<br>-/+
	Male sex	Higher age	History of diabetes mellitus	History of dyslipidaemia	History of hypertension	History of CHD	History of MI	History of AP	Family history of MI	Smoking	Obesity	Central chest pain	Left-sided chest pain	Radiation to left arm/ shoulder	Visceral pain	Stabbing pain	Typical angina	Sweating	Dyspnoea	Nausea/ vomiting	Palpitations
Reference disease	**< 0.001**<br>+/++	0.99<br>+	**<0.01**<br>+/++	0.89<br>+	**0.12**<br>+/++	**< 0.001**<br>+/++	**< 0.001**<br>+/++	**0.02**<br>+/++	0.54<br>-	0.31<br>+	0.90<br>-	0.96<br>-	*<br>+	0.31<br>+/++	0.49<br>-	0.61<br>-	1.00<br>-	0.93<br>-	0.90<br>-	*<br>-	0.96<br>-/+
Reference standard	**< 0.001**<br>+/++	0.99<br>+	**0.03**<br>+	0.45<br>-	0.94<br>-	**< 0.001**<br>+/++	**< 0.001**<br>+/++	0.29<br>+	0.47<br>-	0.95<br>-	**0.07**<br>+/++	0.69<br>-/+	0.30<br>+	**0.02**<br>+/++	0.94<br>+	0.31<br>+	**0.05**<br>-	*<br>+	**0.09**<br>-	0.79<br>-	1.00<br>-/+
Setting	0.99<br>-/+	0.55<br>+	0.90<br>-	0.90<br>-	0.75<br>-	0.99<br>-	0.99<br>-/+	0.85<br>-	0.84	0.88<br>-	cons	Cons.	Cons.	Cons.	Cons.	Cons.	0.58<br>-	0.95<br>-	0.96<br>-/+	0.94<br>-/+	0.95<br>-
Selection?	**0.08**<br>+	1.00<br>-	0.54<br>-	0.18<br>-	0.93<br>-	0.87<br>-	0.79<br>-	0.11<br>+	0.82<br>-	0.84<br>-	0.59<br>-	0.99.<br>+	0.92<br>-	0.14<br>-	0.88<br>-/+	0.58<br>-	**0.002**<br>-/+	0.89<br>-	0.16<br>-	0.47<br>-	0.14<br>-
Definite sick excluded?	0.76<br>-	0.90<br>-/+	0.81	0.95<br>-	0.42<br>-	0.98<br>+	1.00<br>-	cons.	0.38<br>-/+	0.98<br>-	0.22<br>+	0.61<br>-	0.54<br>-	0.53<br>-	0.99<br>-	0.73<br>-	0.94<br>-	Cons.	1.00<br>-	Cons.	1.00<br>-
Pain acute-chronic	**0.01**<br>+/++	0.99<br>-	0.18<br>+/++	0.95<br>-	0.57<br>-	**< 0.001**<br>+/++	**< 0.001**<br>+/++	1.00<br>-	0.72<br>+	0.41<br>-	0.64<br>-	0.72<br>-/+	0.57<br>+	*<br>+/++	0.82<br>-/+	0.97<br>-	0.95<br>-/+	0.99<br>-	0.42<br>-	0.97<br>-	0.14<br>-
Prevalence	0.15<br>-	0.51<br>-	0.47<br>-	0.50<br>+	0.84<br>-	1.00<br>+	0.96<br>-	0.47<br>-/+	0.11<br>+	0.98<br>-	0.97<br>-	0.95<br>-	0.22<br>+	0.90<br>-/+	0.99<br>-	0.72<br>-	0.99<br>+	*<br>-	0.64<br>-	0.98<br>-	0.11<br>-
Size	0.66	0.94	0.79	0.28	0.82	0.52	0.99	0.65	1.00	0.81	0.28	0.43	0.86	1.00	0.78	0.58	0.79	0.98	0.79	0.79	0.18
	-	-	-	-	-	-	-	-	-	-	-	-	-	-	-	-	-	-	-	-	-
Date of Publication	0.14	1.00	**0.06**	0.94	**0.02**	0.44	0.42	0.16	0.61	**0.01**	1.00	0.78	1.00	0.85	0.96	0.46	**0.01**	0.94	0.94	0.94	0.70

	+/++	-	+/++	-	+/++	-	-	+	-	+/++	-	-	-	-	-	-	+/++	-	-	-	-

Cons.: Covariate was constant; - no impact; + possible impact; ++ probably impact; * bivariate random effects model produced unstable estimates or did not converge. A p value ≤ 0.1 was considered to be significant.

CHD: Coronary heart disease; MI: Myocardial infarction; AP: angina pectoris
