# Supplementary material for: Does the patient with chest pain have a coronary heart disease? Diagnostic value of single symptoms and signs – a meta-analysis
Source: Croat Med J. 2012 Oct;53(5):432–41. doi: 10.3325/cmj.2012.53.432 (PMC3490454; doi:10.3325/cmj.2012.53.432)
Supplement: Supplementary Table 7 [file CroatMedJ_53_s007.pdf]

Supplemental table 7: Subgroup analysis in regard to study quality

Table presents results within subgroups if a quality criterion was significantly associated with the accuracy of an index test (see table 6).

Criterion	Studies (n)	LR (95% CI) if RF is	
		present	absent
	**Male sex**		
All studies	102	1.21 (1.16-1.27)	0.70 (0.65-0.75)
Representative spectrum yes	74	1.17 (1.16-1.23)	0.76 (0.69-0.81)
Representative spectrum no or unclear	28	1.32 (1.21-1.44)	0.58 (0.51-0.66)
Reference results blinded yes	7	1.66 (1.33-2.09)	0.41 (0.32-0.52)
Reference results blinded no or unclear	95	1.19 (1.14-1.24)	0.72 (0.67-0.77)
	**History of dyslipidaemia**		
All studies	46	1.52 (1.34-1.73)	0.73 (0.67-0.80)
Representative spectrum yes	32	1.40 (1.22-1.61)	0.78 (0.72-0.86)
Representative spectrum no or unclear	14	1.82 (1.44-2.31)	0.61 (0.72-0.86)
	**History of MI**		
All studies	52	1.37 (1.14-1.64)	0.87 (0.80-0.95)
Representative spectrum yes	42	1.98 (1.34-2.92)	0.67 (0.53-0.84)
Representative spectrum no or unclear	10	1.25 (1.02-1.52)	0.91 (0.84-0.98)
	**Family history of MI**		
All studies	34	1.19 (1.06-1.33)	0.90 (0.85-0.96)
Differential verification avoided yes	25	1.09 (0.98-1.21)	0.94 (0.88-1.01)
Differential verification avoided no or unclear	9	1.58 (1.25-2.00)	0.83 (0.75 (0.92)
	**Obesity**		
All studies	12	1.06 (0.88-1.26)	0.97 (0.89-1.07)
Withdrawals explained? yes	4	0.73 (0.49-1.19)	1.06 (0.96-1.18)
Withdrawals explained no or unclear	8	1.10 (0.92-1.31)	0.93 (0.82-1.05
Differential verification avoided yes	7	1.09 (0.90-1.31)	0.93 (0.81-1.08)
Differential verification avoided no or unclear	5	0.94 (0.63-1.37)	1.02 (0.92-1.12)
	**Left-sided chest pain**		
All studies		0.85 (0.60-1.20)	1.06 (0.96-1.18)
Partial verification avoided yes	9	0.70 (0.50-0.99)	1.10 (1.02-1.18)
Partial verification avoided no or unclear	3	1.31 (0.99-1.73)	0.74 (0.49-1.12)
	**Radiation to left arm**		
All studies	12	1.30 (1.12-1.52)	0.86 (0.78-0.95)
Representative spectrum yes	9	1.42 (1.27-1.60)	0.82 (0.75-0.90)
Representative spectrum no or unclear	3	0.94 (0.78-1.14)	1.04 (0.92-1.17)
	**Visceral pain**		
All studies	17	1.34 (1.07-1.67)	0.78 (0.60-1.02)
Differential verification avoided yes	9	0.96 (0.68-1.33)	1.03 (0.85-1.24)
Differential verification avoided no or unclear	8	1.70 (1.43-2.02)	0.50 (0.33-0.77)

LR: likelihood ratio
