# Supplementary material for: Does the patient with chest pain have a coronary heart disease? Diagnostic value of single symptoms and signs – a meta-analysis
Source: Croat Med J. 2012 Oct;53(5):432–41. doi: 10.3325/cmj.2012.53.432 (PMC3490454; doi:10.3325/cmj.2012.53.432)
Supplement: Supplementary Table 8 [file CroatMedJ_53_s008.pdf]

Supplemental table 8: Results of primary studies for the accuracy of 1 risk factor in which BREM did not produce stable estimates.

Case definition of CHD	Studies	Patients (n)	LR (95% CI) If RF is present	LR (95% CI) If RF absent
	**Menopause**			
Stable CHD	Warner 1995	55	1.18 (0.79-1.78)	0.75 (0.36-1.56)
Stable CHD	Sharaf 2001	323	1.12 (1.00-1.26)	0.65 (0.42-1.01)
Stable CHD	Sanfilippo 2005	158	1.12 (0.93-1.36)	0.65 (0.28-1.52)
Stable CHD	Johnson 2006	673	1.11 (1.02-1.21)	0.70 (0.52-0.94)
Stable CHD	Shaw 2006	883	1.15 (1.07-1.24)	0.62 (0.48-0.81)

BREM: bivariate random effects model ; CHD: coronary heart disease; LR: likelihood ratio
