# Supplementary material for: Does the patient with chest pain have a coronary heart disease? Diagnostic value of single symptoms and signs – a meta-analysis
Source: Croat Med J. 2012 Oct;53(5):432–41. doi: 10.3325/cmj.2012.53.432 (PMC3490454; doi:10.3325/cmj.2012.53.432)
Supplement: Supplementary Table 9 [file CroatMedJ_53_s009.pdf]

Supplemental table 9: Results of primary studies for the accuracy of 5 pain characteristics in which BREM (with and/ or without 'case definition of CHD' as covariate) did not produce stable estimates.

Case definition of CHD	Studies	Patients (n)	LR (95% CI)	
			If RF is present	If RF absent
	**Left-sided chest pain**			
Stable CHD	Davies 1985	100	3.02 (1.74-5.23)	0.40 (0.26-0.62)
Stable CHD	Mukerji 1989	246	0.58 (0.35-0.96)	1.16 (1.01-1.32)
Stable CHD	Cooke 1997	130	0.60 (0.15-2.41)	1.03 (0.95-1.13)
Stable CHD	Sanfilippo 2005	158	0.67 (0.28-1.57)	1.11 (0.92-1.34)
Stable CHD	Wu 2005	405	1.03 (0.65-1.63)	0.99 (0.91-1.08)
MI	Sawe 1971	132	1.02 (0.63-1.65)	0.99 (0.76-1.28)
MI	Sawe 1972a	191	0.54 (0.28-1.02)	1.14 (0.99-1.30)
MI	Beunderman 1983	106	1.03 (0.77-1.38)	0.95 (0.58-1.57)
MI	Tierney 1986	492	0.11 (0.02-0.77)	1.16 (1.10-1.22)
MI	Everts 1996	903	1.12 (0.97-1.30)	0.91 (0.81-1.03)
MI	Albarran 2002	541	1.03 (0.87-1.23)	0.97 (0.82-1.14)
MI	Baxt 2002	2204	0.83 (0.64-1.07)	1.11 (0.98-1.26)
	**Radiation to left arm/ shoulder**			
Stable CHD	Davies 1985	100	1.54 (0.85-2.80)	0.82 (0.63-1.07)
Stable CHD	Cooke 1997	130	1.00 (0.72-1.39)	1.00 (0.70-1.43)
Stable CHD	Wu 2005	405	0.86 (0.66-1.12)	1.09 (0.94-1.27)
Stable CHD	Shaw 2006	883	0.92 (0.76-1.12)	1.04 (0.95-1.15)
MI	Sawe 1971	132	1.49 (0.81-2.74)	0.88 (0.71-1.08)
MI	Sawe 1972a	191	1.14 (0.70-1.86)	0.96 (0.81-1.13)
MI	Berger 1990	278	2.28 (1.66-3.12)	0.59 (0.47-0.75)
MI	Gaston-Johansson 1991	94	1.61 (1.07-2.41)	0.61 (0.38-0.97)
MI	Everts 1996	903	1.23 (1.07-1.42)	0.84 (0.74-0.95)
MI	Baxt 2002	2204	1.42 (1.10-1.83)	0.87 (0.77-0.99)
ACS	Christenson 2006	870	1.53 (1.30-1.81)	0.68 (0.57-0.82)
ACS	Han 2007	10126	1.33 (1.21-1.46)	0.87 (0.83-0.92)
	**Radiation to back**			
Stable CHD	Cooke 1997	130	1.05 (0.62-1.78)	0.98 (0.78-1.23)
Stable CHD	Wu 2005	405	0.48 (0.28-0.81)	1.11 (1.03-1.21)
MI	Sawe 1972a	191	0.73 (0.37-1.43)	1.06 (0.93-1.20)
MI	Gaston-Johansson 199	94	0.65 (0.32-1.35)	1.16 (0.91-1.48)
MI	Albarran 2002	541	0.71 (0.54-0.95)	1.13 (1.02-1.26)
	**Frightening pain**			
MI	Hofgren 1994	899	0.90 (0.74-1.10)	1.04 (0.96-1.14)
MI	Gaston-Johansson 1999	94	14.87 (0.84-264.3)	0.87 (0.77-0.99)
MI	Albarran 2000	267	1.21 (0.87-1.67)	0.90 (0.75-1.08)
ACS	Jerlock 2007	348	0.71 (0.30-1.68)	1.06 (0.93-1.21)
	**Crescendo angina**			
Stable CHD	Davies 1985	100	1.85 (0.87-3.92)	0.83 (0.67-1.04)
Stable CHD	Sanfilippo 2005	158	1.38 (1.01-1.88)	0.65 (0.38-1.10)
MI	Schroeder 1979	193	0.99 (0.79-1.25)	1.01 (0.71-1.43)
	**Pain related to breathing**			
Stable CHD	Vodopiutz 2002	92	0.36 (0.17-0.76)	1.52 (1.14-2.02)
Stable CHD	Wu 2005	405	0.22 (0.04-1.08)	1.03 (1.00-1.06)
MI	Solomon 1989	7734	0.20 (0.15-0.27)	1.18 (1.16-1.20)

BREM: bivariate random effects model; CHD: coronary heart disease; MI: myocardial infarction; ACS: acute coronary syndrome; LR: likelihood ratio
