# Supplementary material for: Does the patient with chest pain have a coronary heart disease? Diagnostic value of single symptoms and signs – a meta-analysis
Source: Croat Med J. 2012 Oct;53(5):432–41. doi: 10.3325/cmj.2012.53.432 (PMC3490454; doi:10.3325/cmj.2012.53.432)
Supplement: Supplementary Table 10 [file CroatMedJ_53_s010.pdf]

Supplemental table 10: Results of primary studies for the accuracy of 6 associated symptoms in which BREM (with and/ or without 'case definition of CHD' as covariate) did not produce stable estimates.

Case definition of CHD	Studies	Patients (n)	LR (95% CI)	
			**If symptom is present**	**If symptom absent**
	**Sweating**			
MI	Short 1981	456	2.66 (0.91-7.81)	0.97 (0.93-1.00)
MI	Beunderman 1983	106	1.33 (0.77-2.31)	0.87 (0.66-1.14)
MI	Logan 1986	180	1.47 (1.18-1.83)	0.43 (0.27-0.69)
MI	Tierney 1986	492	4.62 (2.67-8.00)	0.77 (0.66-0.90)
MI	Solomon 1989	7734	1.97 (1.84-2.10)	0.64 (0.60-0.68)
MI	Jonsbu 1991	200	2.97 (1.97-4.49)	0.54 (0.41-0.71)
MI	Herlitz 1995	491	1.53 (1.01-2.32)	0.90 (0.80-1.01)
MI	Baxt 2002	2204	1.95 (1.56-2.44)	0.74 (0.64-0.86)
MI	Svensson 2003	536	1.91 (1.55-2.36)	0.62 (0.52-0.75)
MI	Bulgiba 2005	620	1.55 (1.38-1.74)	0.42 (0.31-0.56)
ME	Miller 2004	2992	1.40 (0.98-2.00)	0.90 (0.79-1.03)
	**Nausea/ vomiting**			
Stable CHD	Davies 1985	100	0.79 (0.29-2.19)	1.04 (0.89-1.21)
MI	Short 1981	456	1.52 (0.99-2.33)	0.92 (0.85-1.00)
MI	Beunderman 1983	106	2.44 (1.24-4.80)	0.70 (0.54-0.91)
MI	Logan 1986	180	1.70 (1.22-2.38)	0.62 (0.46-0.82)
MI	Tierney 1986	492	1.86 (1.43-2.43)	0.63 (0.47-0.84)
MI	Herlihy 1987	265	1.91 (1.40-2.61)	0.65 (0.52-0.80)
MI	Berger 1990	278	2.52 (1.67-3.78)	0.70 (0.59-0.84)
MI	Jonsbu 1991	200	1.98 (1.32-2.98)	0.71 (0.56-0.89)
MI	Herlitz 1995	491	1.23 (0.82-1.82)	0.95 (0.84-1.06)
MI	Baxt 2002	2204	1.28 (0.95-1.73)	0.93 (0.83-1.03)
MI	Svensson 2003	536	1.40 (1.10-1.78)	0.83 (0.72-0.96)
MI	Bulgiba 2005	620	1.38 (1.15-1.64)	0.77 (0.65-0.90)
ACS	Pope 1998	10154	1.11 (1.04-1.19)	0.96 (0.93-0.99)
	**Dizziness**			
Stable CHD	Vodopiutz 2002	92	0.49 (0.25-0.95)	1.38 (1.04-1.84)
Stable CHD	Wu 2005	405	0.47 (0.33-0.66)	1.31 (1.15-1.49)
MI	Beunderman 1983	106	0.72 (0.45-1.15)	1.25 (0.91-1.72)
MI	Hartford 1993	226	0.70 (0.42-1.18)	1.10 (0.96-1.26)
MI	Herlitz 1995	491	0.43 (0.16-1.19)	1.05 (1.00-1.11)
MI	Hofgren 1995	914	0.62 (0.32-1.18)	1.02 (0.99-1.05)
ACS	Pope 1998	9222	0.61 (0.56-0.67)	1.17 (1.14-1.20)
	**Collapse**			
MI	Sawe 1972	921	0.87 (0.68-1.12)	1.04 (0.97-1.11)
MI	Sawe 1972a	191	0.66 (0.40-1.09)	1.14 (0.97-1.35)
MI	Short 1981	456	1.18 (0.48-2.94)	0.99 (0.96-1.03)
MI	Herlitz 1995	491	0.82 (0.28-2.36)	1.01 (0.97-1.05)
MI	Hofgren 1995	914	1.82 (0.62-5.40)	0.99 (0.98-1.01)
ACS	Pope 1998	8920	0.25 (0.19-0.34)	1.06 (1.06-1.07)
ACS	Herlitz 2002	921	0.31 (0.17-0.56)	1.10 (1.06-1.15)
ACS	Soiza 2005	869	0.32 (0.21-0.48)	1.14 (1.08-1.21)
ACS	Myint 2007	187	0.42 (0.26-0.67)	1.53 (1.24-1.89)
	**Weakness**			
MI	Schroeder 1979	193	1.09 (0.72-1.63)	0.96 (0.79-1.17)
MI	Beunderman 1983	106	1.05 (0.67-1.63)	0.97 (0.70-1.34)
MI	Logan 1986	180	1.32 (1.06-1.64)	0.57 (0.37-0.88)
MI	Hartford 1993	226	0.86 (0.56-1.32)	1.06 (0.90-1.24)
MI	Hofgren 1995	914	0.97 (0.79-1.19)	1.01 (0.93-1.10)
ME	Miller 2004	2992	1.71 (1.28-2.29)	0.81 (0.69-0.95)
	**Fear/ anxiety**			
Stable CHD	Mayou 1994	90	1.10 (0.82-1.49)	0.83 (0.46-1.49)
Stable CHD	Fraenkel 1996	114	0.36 (0.20-0.65)	1.64 (1.21-2.22)
MI	Beunderman 1983	106	0.84 (0.63-1.12)	1.38 (0.82-2.31)

BREM: bivariate random effects model ; CHD: coronary heart disease; MI: myocardial infarction; ACS: acute coronary syndrome; ME:

major cardiac event; LR: likelihood ratio
