# Supplementary material for: Does the patient with chest pain have a coronary heart disease? Diagnostic value of single symptoms and signs – a meta-analysis
Source: Croat Med J. 2012 Oct;53(5):432–41. doi: 10.3325/cmj.2012.53.432 (PMC3490454; doi:10.3325/cmj.2012.53.432)
Supplement: Supplementary Table 11 [file CroatMedJ_53_s011.pdf]

Supplemental table 11: Results of primary studies for the accuracy of 6 signs in which BREM (with and/ or without ‘case definition of CHD’ as covariate) did not produce stable estimates.

Case definition of CHD	Studies	Patients (n)	LR (95% CI)	
			If sign is present	If sign is absent
	**High blood pressure**			
Stable CHD	Cooke 1997	130	7.33 (2.31-23.31)	0.69 (0.58-0.83)
MI	Sawe 1972a	191	0.85 (0.64-1.13)	1.18 (0.88-1.58)
MI	Herlitz 2002a	1592	0.90 (0.63-1.28)	1.01 (0.97-1.06)
	**Tachycardia**			
MI	Sawe 1972a	191	1.14 (0.72-1.81)	0.95 (0.80-1.13)
MI	van der Does 1980	1306	20.50 (12.37-33.96)	0.66 (0.56-0.76)
ACS	Bjork 2006	632	1.29 (0.42-3.93)	0.99 (0.96-1.03)
	**Bradycardia**			
MI	Sawe 1972a	191	1.96 (0.64-6.04)	0.95 (0.88-1.03)
MI	van der Does 1980	1306	13.04 (7.28-23.36)	0.80 (0.72-0.89)
ACS	Bjork 2006	632	1.16 (0.32-4.15)	1.00 (0.97-1.03)
	**Rales**			
MI	Sawe 1972a	191	1.65 (1.04-2.63)	0.81 (0.67-0.98)
MI	van der Does 1980	1306	13.70 (7.71-24.34)	0.79 (0.71-0.88)
MI	Sirnes 1984	222	0.70 (0.50-0.97)	1.29 (0.99-1.68)
MI	Eriksson 1994	80	0.90 (0.41-1.98)	1.03 (0.81-1.32)
MI	Baxt 2002	2204	2.18 (1.51-3.16)	0.88 (0.80-0.96)
ACS	Pope 1998	10387	1.26 (1.16-1.37)	0.94 (0.92-0.96)
ACS	Green 2006	634	2.02 (1.03-3.96)	0.95 (0.90-1.01)
ME	Goldman 1996	4676	1.78 (1.37-2.31)	0.87 (0.79-0.95)
	**Pain reproducible by palpation**			
MI	Lee 1985	596	0.25 (0.13-0.50)	1.33 (1.23-1.44)
MI	Tierney 1986	492	0.41 (0.22-0.76)	1.33 (1.17-1.51)
MI	Solomon 1989	7734	0.21 (0.15-0.28)	1.16 (1.14-1.18)
MI	Lee 1990	5674	0.21 (0.15-0.30)	1.17 (1.15-1.19)
MI	Lopez-Jimenez 1998	2694	0.29 (0.18-0.45)	1.37 (1.30-1.45)
MI	Goodacre 2002	893	0.32 (0.11-0.94)	1.26 (1.13-1.41)
ME	Miller 2004	2992	0.13 (0.03-0.53)	1.18 (1.14-1.23)

BREM: bivariate random effects model; CHD: coronary heart disease; MI: myocardial infarction; ACS: acute coronary syndrome; ME: major cardiac event; LR: likelihood ratio
